# Mechanical Intercellular Communication via Matrix‐Borne Cell Force Transmission During Vascular Network Formation

**DOI:** 10.1002/advs.202306210

**Published:** 2023-11-23

**Authors:** Christopher D. Davidson, Firaol S. Midekssa, Samuel J. DePalma, Jordan L. Kamen, William Y. Wang, Danica Kristen P. Jayco, Megan E. Wieger, Brendon M. Baker

**Affiliations:** ^1^ Department of Biomedical Engineering University of Michigan Ann Arbor MI 48109 USA; ^2^ Department of Chemical Engineering University of Michigan Ann Arbor MI 48109 USA

**Keywords:** calcium signaling, electrospinning, endothelial cells, extracellular matrix, fibrous matrices, focal adhesion kinase, focal adhesions, force transmission, mechanical communication, mechanosensitive ion channels, Piezo1, TRPV4, vasculogenic assembly

## Abstract

Intercellular communication is critical to the formation and homeostatic function of all tissues. Previous work has shown that cells can communicate mechanically via the transmission of cell‐generated forces through their surrounding extracellular matrix, but this process is not well understood. Here, mechanically defined, synthetic electrospun fibrous matrices are utilized in conjunction with a microfabrication‐based cell patterning approach to examine mechanical intercellular communication (MIC) between endothelial cells (ECs) during their assembly into interconnected multicellular networks. It is found that cell force‐mediated matrix displacements in deformable fibrous matrices underly directional extension and migration of neighboring ECs toward each other prior to the formation of stable cell‐cell connections enriched with vascular endothelial cadherin (VE‐cadherin). A critical role is also identified for calcium signaling mediated by focal adhesion kinase and mechanosensitive ion channels in MIC that extends to multicellular assembly of 3D vessel‐like networks when ECs are embedded within fibrin hydrogels. These results illustrate a role for cell‐generated forces and ECM mechanical properties in multicellular assembly of capillary‐like EC networks and motivates the design of biomaterials that promote MIC for vascular tissue engineering.

## Graphical abstract

This work describes how endothelial cells (ECs) utilize mechanical intercellular communication (MIC) during vasculogenic assembly into capillary‐like, multicellular networks. Mechanically permissive matrices and EC actomyosin activity enable long‐range force transmission to direct cell migration and subsequent cell‐cell contact formation. In particular, focal adhesion signaling and stretch‐induced activation of ion channels (Piezo1/TRPV4) are critical to EC MIC and vasculogenic assembly.

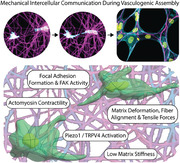

## 1 Introduction

The ability of cells to communicate and coordinate their activity is crucial to the development and homeostatic function of all tissues, and as such, holds high relevance to tissue engineering.^[^ ^1^ ^]^ Intercellular communication through receptor‐ligand interactions at cell‐cell interfaces or via diffusive soluble factors has been extensively studied.^[^ ^2^, ^3^, ^4^, ^5^ ^]^ In addition to these well‐established means of biochemically mediated intercellular signaling, a more recent body of evidence has shown that cells can also communicate via cell‐generated forces transmitted to neighboring cells through the extracellular matrix (ECM), which we term mechanical intercellular communication (MIC).^[^ ^6^, ^7^ ^]^ Mechanical interactions between cells and the ECM are important in various processes such as cell spreading, migration and tissue morphogenesis.^[^ ^8^, ^9^, ^10^, ^11^ ^]^ Cells engage and apply forces to their surrounding matrix through integrin‐based adhesion complexes, or focal adhesions (FAs), which mechanically connect the ECM to the cell's actomyosin cytoskeleton.^[^ ^12^ ^]^ FAs serve as mechanochemical signaling hubs, allowing cells to continuously sense passive mechanical and topographical properties of the matrix as well as active external forces applied to the cell.^[^ ^13^ ^]^ Concurrently, cell‐generated forces applied to the ECM through FAs result in matrix deformations that may impact surrounding cells. The dynamic and reciprocal nature of generating and sensing mechanical signals, however, makes MIC intrinsically difficult to investigate. Specifically, we have a limited understanding of the cellular machinery required for cells to sense and respond to tensile forces originating from neighboring cells. Further, how tissue‐relevant matrix properties mediate the transmission of cell‐generated forces has not been well‐established.

EC interactions with their microenvironment through sensing and responding to mechanical forces and biochemical stimuli are vital for vascular assembly and homeostasis.^[^ ^14^ ^]^ Mechanosensing is prevalent in ECs undergoing vasculogenic assembly and angiogenesis during the creation of microvasculature, where numerous studies have reported that matrix stiffness, topography, and curvature affect EC behavior.^[^ ^14^ ^]^ Similarly, mechanosensing plays a prominent role in the maintenance of vascular function in the endothelium. The endothelium, due to its position between the vascular wall and the bloodstream, is constantly exposed to a complex set of mechanical forces that are highly dynamic in nature.^[^ ^14^ ^]^ As such, the inability of the endothelium to appropriately perceive and respond to various biomechanical stimuli has been implicated in numerous pulmonary and cardiovascular pathologies.^[^ ^14^, ^15^, ^16^, ^17^ ^]^

How MIC coordinates the self‐assembly of ECs into multicellular networks during vasculogenic assembly is not understood and thus the focus of this work.^[^ ^11^ ^]^ Previous attempts to study the implications of matrix mechanical properties in vasculogenic assembly have primarily focused on modulating the elastic modulus of natural bulk hydrogels (eg. Matrigel, fibrin, collagen).^[^ ^18^, ^19^ ^]^ However, due to the cross‐dependence of matrix mechanical properties, topography, stiffness, and ligand density as a function of ECM protein concentration within these hydrogels, discerning the specific contribution of biochemical and biophysical signals has proven challenging.^[^ ^19^, ^20^, ^21^ ^]^ To address this, many groups have utilized modular and orthogonal design strategies to generate synthetic hydrogels modified with ECM proteins or peptides.^[^ ^22^ ^]^ While these strategies have been insightful in isolating the contribution of biochemical and biophysical signals in vascular network assembly, many synthetic hydrogels are non‐fibrous, elastic materials, that are mechanically quite distinct from native fibrous ECM.^[^ ^23^, ^24^ ^]^ As such, the relationship between fibrous matrix mechanics, MIC, and vasculogenic assembly has not previously been explored. Intriguingly, several computational modeling approaches suggest that fibrous matrices are optimal for transmitting forces over large distances (i.e., greater than one cell body away) due to their nonlinear elastic behavior and the potential for strain‐induced alignment of ECM fibers.^[^ ^25^, ^26^, ^27^, ^28^, ^29^, ^30^, ^31^, ^32^, ^33^, ^34^ ^]^ Our lab has previously developed synthetic matrices of electrospun dextran‐based hydrogel fibers with user‐defined architecture and mechanical properties.^[^ ^35^, ^36^, ^37^, ^38^, ^39^ ^]^ Here, we combined this biomaterial approach with a microfabrication‐based cell‐patterning method to examine how biophysical properties of fibrous ECM regulate MIC between endothelial cells (ECs) during multicellular assembly and investigate the cellular machinery involved.

## 2 Results

### 2.1 Cell‐Generated Matrix Deformations and Tension Promote Enhanced Cell Spreading and Multicellular Cluster Formation

**Figure 1:**
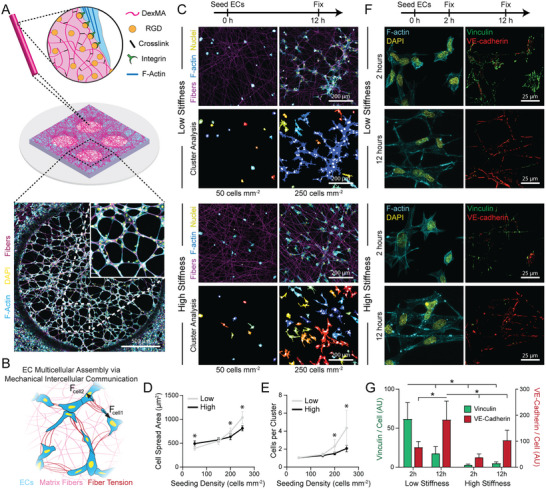
Cell‐generated matrix deformations and tension enhance cell spreading and multicellular cluster formation. (A) Schematic of microfabricated PDMS multi‐well substrate containing an array of wells, each supporting an isolated suspended matrix of DexMA fibers functionalized with RGD to facilitate cell adhesion. (B) Schematic of hypothesis that matrix fibers enable MIC to facilitate EC network formation. (C) Confocal fluorescent images of phalloidin‐stained ECs (cyan), nuclei (yellow), and rhodamine‐labeled DexMA fibers (magenta) with respective color‐coded maps of contiguous actin clusters at low (50 cells mm^−2^) and high (250 cells mm^−2^) seeding density in low stiffness, cell‐deformable matrices and high stiffness, non‐deformable matrices. (D) Quantification of cell spread area and (E) average number of ECs per contiguous actin cluster as a function of seeding density and matrix stiffness (*n* = 12 fields of view). (F) Confocal fluorescent images of phalloidin‐stained ECs (cyan), nuclei (yellow), vinculin (green), and VE‐cadherin (red) at 2 and 12 hours after seeding in low stiffness and high stiffness matrices. (G) Quantification of total vinculin and VE‐cadherin fluorescent intensity normalized to cell density as a function of time and matrix stiffness (n = 6 fields of view). All data presented as mean ± SD; asterisk denotes significance with *p* < 0.05.

A deeper understanding of long‐range MIC in the context of vasculogenic assembly could inform the improved design of vascularized biomaterials.^[^ ^40^ ^]^ Vasculogenic assembly involves the directed self‐assembly of dispersed, individual ECs into an interconnected network of capillary‐like structures and requires cellular communication and coordination over length‐scales far larger than that of a cell.^[^ ^41^, ^42^ ^]^ If better understood, pharmacologic or material control over this process presents a promising approach to engineer microvasculature to support parenchymal cells, a major challenge in the field of tissue engineering and regenerative medicine.^[^ ^43^ ^]^ We previously found that cell force‐mediated matrix reorganization portends EC network assembly, implicating matrix stiffness in MIC.^[^ ^36^ ^]^ To more closely examine mechanical interactions between ECs as a function of fibrous matrix stiffness, populations of ECs were heterogeneously seeded onto matrices of electrospun dextran methacrylate (DexMA) fibers with tunable stiffness suspended over an array of microfabricated wells (**Figure** **1**A).^[^ ^35^ ^]^

To test our hypothesis that cell forces and resulting matrix deformations mediate MIC between ECs (Figure 1B), we first seeded cells over a range of densities on low stiffness matrices that were deformable (E = 0.724 kPa) or high stiffness matrices that were non‐deformable (E = 19.7 kPa) under EC traction forces (Figure S1, Supporting Information). As seeding density inversely correlates with the average distance between neighboring cells, we hypothesized the effect of MIC would manifest as cell‐density dependent differences in cell spreading on deformable versus non‐deformable matrices. At low seeding densities (50 cells mm^−2^), isolated cells remained largely unspread independent of matrix stiffness, although a moderately higher spread area was noted in stiff, non‐deformable matrices (Figure 1C,D). However, increasing seeding density resulted in more marked increases in cell spread area in deformable matrices as compared to non‐deformable matrices, indicating a synergistic influence of matrix stiffness and seeding density (cell proximity) on cell spreading (Figure 1D). Along with this increase in spread area, larger clusters of interconnected cells formed on soft, cell‐deformable matrices in contrast to equivalent cell seeding densities on stiff, non‐deformable matrices (Figure 1E). Although this experiment did not control for paracrine effects which likely are operative, these results suggest that the distance between cells and the resulting sensing of cell force‐mediated matrix displacements can influence cell spreading and the formation of interconnected, multicellular clusters.

We next investigated cell‐ECM and cell‐cell adhesion during this process by immunostaining for vinculin (a force‐sensitive component of FAs) and VE‐cadherin (the direct linkage between ECs at adherens junctions), respectively (Figure 1F). At an early time point when cells are putatively sending and receiving mechanical signals (2 h post‐seeding), we observed significantly more vinculin‐rich FAs in deformable compared to non‐deformable matrices, suggesting heightened cell‐ECM adhesion and force transmission to the matrix. Additionally, at 12 h post‐seeding when multicellular clusters had emerged, we observed significantly heightened VE‐cadherin localization to cell‐cell junctions in deformable matrices compared to non‐deformable matrices (Figure 1G). Together, these results indicate that cell‐deformable fibrous matrices facilitate cell‐ECM adhesions that presage the formation of robust adherens junctions and overall, provide motivation for the involvement of cell‐generated forces transmitted between cells through the matrix to mediate MIC.

To investigate the dynamics of EC assembly into multicellular clusters, timelapse imaging of Hoechst‐labeled ECs lentivirally transduced with an F‐actin reporter (LifeAct‐GFP) was conducted over the same 12 h timeframe spanning cell spreading and network assembly. As in the previous experiment, ECs actively recruited matrix fibers and formed large multicellular clusters in deformable matrices, a phenomenon not observed in non‐deformable matrices (Movie S1, Supporting Information). ECs additionally migrated overall faster in deformable matrices compared to in non‐deformable matrices (Figure S2A–C, Supporting Information). Interestingly, enhanced migration speeds over the first two hours in deformable matrices coincided with a rapid increase in multicellular cluster size (Figure S2D, Supporting Information). We noted instances of directed extension and migration of neighboring cells towards each other to form cell‐cell contacts and multicellular clusters in deformable matrices, while migration in non‐deformable matrices appeared uncoordinated and random (Figure S2E and Movie S1, Supporting Information). Together, this data suggests that ECs are capable of responding to cell‐generated forces transmitted through cell‐deformable ECM by extending towards one another and/or directionally migrating to form multicellular structures.

### 2.2 Micropatterning Single ECs Reveals Matrix Stiffness Influences Cell Spreading, FA Formation, and Matrix Deformations

**Figure 2:**
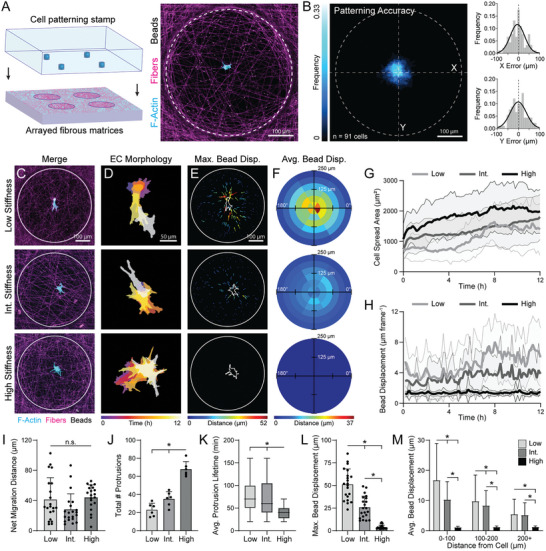
Micropatterning single ECs reveals matrix stiffness influences cell spreading, FA formation, and matrix deformations. A) Schematic depicting microfabrication‐based patterning approach to isolate individual ECs at the center of suspended fibrous matrices. Representative confocal fluorescent image of patterned EC (cyan), rhodamine‐labeled fibers (magenta), and fluorescent microspheres embedded in matrix fibers (white). B) F‐actin heat map of patterned ECs with histograms of average patterning error in x‐ and y‐directions (*n* = 91 cells). C) Representative confocal fluorescent image of LifeAct‐GFP expressing ECs (cyan), rhodamine‐labeled DexMA fibers (magenta), and fiber‐embedded fluorescent microspheres (white). D) Temporally color‐coded overlay of EC cell bodies over a 12‐hour time course following patterning. E) Size‐ and color‐coded vector plots displaying maximum displacement of each microsphere over a 12‐hour time course. F) Binned average microsphere displacements for all ECs aligned along their long axis (0°) with color‐coded magnitudes (*n* > 20 cells). G) Cell spread area and H) fluorescent microsphere displacement over a 12‐hour time course as a function of matrix stiffness (*n* > 20 cells). (I) Net migration distance (*n* > 20 cells), J) total number of protrusions (*n* = 6 cells), K) average protrusion lifetime (*n* = 6 cells, *n* = 30 protrusions), and L) maximum microsphere displacement as a function of matrix stiffness (*n* > 20 cells). M) Binned average microsphere displacements as a function of starting distance from the cell centroid (*n* > 20 cells, *n* > 627 microspheres). All data presented as mean ± SD with superimposed data points; asterisk denotes significance with *p* < 0.05.

While the previous model provides evidence for MIC in deformable fibrous matrices, the highly dynamic and reciprocal nature of generating, receiving, and responding to mechanical signals within a heterogeneously distributed population of cells is challenging to dissect. Specifically, heterogeneous cell seeding in this setting precludes measuring isolated strain fields of individual ECs, which could provide insight into the generation and transmission of mechanical signals. Thus, we developed a microfabrication‐based cell patterning method to precisely pattern individual ECs at the center of a suspended DexMA fiber matrix with defined physical and biochemical properties (**Figure** **2**A,B; Figure S3, Supporting Information).^[^ ^44^ ^]^

Single ECs were patterned in low stiffness/cell‐deformable (E = 0.724 kPa), intermediate stiffness (E = 3.15 kPa), or high stiffness/non‐deformable (E = 19.7 kPa) DexMA matrices, cultured for 12 h, and analyzed for cell spread area (via F‐actin) and FAs (via vinculin). With increasing matrix stiffness, we observed a slight increase in cell spread area (Figure S4A,B, Supporting Information) in agreement with our previous observations at low EC seeding density (Figure 1E, 50 cells mm^−2^). Interestingly, analyzing the number and size of FAs, we observed that ECs in deformable matrices possessed significantly higher average FA area despite a lower total number of adhesions (Figure S4B,C, Supporting Information). This was attributed to a higher proportion of large adhesions (> 3 µm^2^) in low stiffness matrices (8.5%) compared to in intermediate (2.5%) and high (2.7%) (Figure S4D, Supporting Information). Interestingly, the difference in FA area as a function of stiffness when ECs were isolated as single cells is modest compared to the difference noted within a population of cells with bulk seeding (Figure 1), suggesting that the robust FAs formed in low stiffness matrices are a result of active mechanical signals transmitted by neighboring cells within a population of ECs.

We next combined our cell/ECM patterning technique with timelapse confocal microscopy to better capture the dynamic interplay between cell spreading and ECM deformations (Movie S2, Supporting Information). The dynamics of cell spreading and matrix deformations varied with stiffness, specifically in terms of cell spread area as well as the number and lifetime of cell protrusions (Figure 2J,K). In cell‐deformable matrices, ECs generally remained unspread for the first four hours of culture during which cells actively recruited matrix fibers beneath the cell body; in contrast, ECs in intermediate stiffness and non‐deformable matrices began to spread immediately (Figure 2G). Across all stiffness conditions, migration of isolated cells was limited and did not significantly vary with matrix stiffness (Figure 2I). With increasing matrix stiffness, ECs generated more protrusions over the 12‐hour timelapse (Figure 2J; Figure S5, Supporting Information), while the average lifetime of each protrusion decreased (Figure 2K). As cell protrusions and constituent FAs comprise a critical force generating and sensing apparatus of the cell, these data suggest that softer, more deformable matrices not only promote increased FA area, but also support more directional and longer lasting mechanical signals.

In addition, we confirmed expected differences in EC force‐mediated matrix deformations by tracking fluorescent microspheres embedded within matrix fibers. In low stiffness, deformable matrices, measurable microsphere displacements occurred across the entire suspended matrix (up to 250 µm away from the cell's centroid) (Figure 2E,F,L,M; Figure S6, Supporting Information) and furthermore, matrix deformations temporally correlated with increases in cell spreading (Figure 2G,H). With increasing matrix stiffness, however, the magnitude and range of displacements diminished, where high stiffness matrices displayed negligible displacements across the entire matrix (Figure 2l,m).

In addition to stiffness, we also investigated the effect of matrix fiber density by altering the duration of electrospun fiber collection while maintaining a constant degree of crosslinking (equivalent to the lowest stiffness condition above) (Figure S1, Supporting Information). Paralleling results from increasing fiber stiffness across matrices containing a fixed density of fibers, increasing the density of low stiffness fibers decreased the magnitude and range of displacements, although to a lesser degree (Figure S7 and Movie S3, Supporting Information). Taken together, this data indicates that low stiffness, low density fibrous matrices best support long‐range matrix deformations and prime ECs for directed force generation by promoting the formation of larger FAs and fewer but longer‐lived protrusions.

### 2.3 Force transmission through Aligned Fibers Spanning Neighboring Cells Promotes Directed Migration and the Formation of Cell‐Cell Connections

**Figure 3:**
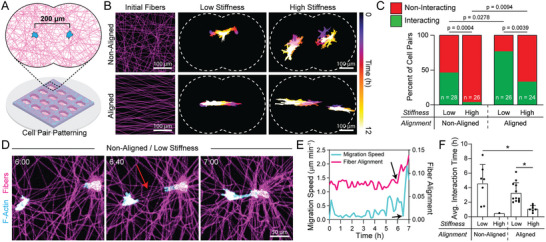
Force transmission through aligned fibers spanning neighboring cells promotes directed migration and the formation of cell‐cell connections. A) Schematic of cell pair patterning in which two ECs are patterned 200 µm apart on a suspended DexMA fibrous matrix. The fiber well is designed such that the distance between the two cells is equal to the distance between cells and well edges. B) Representative image of initial matrix fiber morphology in non‐aligned and aligned conditions. Temporally color‐coded overlays capturing EC morphology and migration over a 12‐hour time course after cell attachment as a function of matrix alignment and stiffness. C) Quantification of the percent of interacting and non‐interacting cells over a 12‐hour time course, with interacting cells defined as cell‐cell contact at any point during the 12‐hour timelapse. P‐values determined by Fisher's exact test. D) Representative cell pair interaction between ECs patterned in a non‐aligned, low stiffness, cell‐deformable matrix, and E) quantification indicating an increase in fiber alignment between the two cells followed by a rapid increase in migration speed prior to cell making direct contact. F) Quantification of average interaction time (duration during which direct cell‐cell contact was maintained). All data presented as mean ± SD with superimposed data points; asterisk denotes significance with *p* < 0.05.

Single cell patterning studies indicate that cell‐deformable, low stiffness matrices prompt ECs to generate larger, more directional matrix displacements, but the generation of a mechanical signal and the associated deformation of the matrix is only an initial step during MIC. Thus, we next investigated how ECs receive and respond to cell‐generated force transmission through fibrous matrices. To do so, we adapted our cell/ECM patterning technique to pattern pairs of ECs at a defined distance 200 µm away from each other (**Figure** **3**A), as our single cell patterning studies demonstrated that ECs in low stiffness matrices generate strain fields measurable 200 µm away from the cell (Figure 2). Combining this approach with timelapse confocal microscopy, we next aimed to determine if and how the stiffness of fibrous matrices regulated intercellular interactions preceding cell‐cell contact.

EC pairs were patterned in cell‐deformable and non‐deformable DexMA matrices and we quantified the frequency that EC pairs formed direct cell‐cell contact at any time over a 12 h period (Movie S4, Supporting Information). In deformable matrices, 46.4% of EC pairs (n = 28) formed direct cell‐cell contact, as compared to 3.9% of EC pairs in high stiffness matrices (*n* = 26) (Figure 3B top row, Figure 3C). Closer examination of EC pairs in deformable matrices revealed alignment of fibers spanning cell pairs that appeared to mediate directional extension, migration, and resulting cell‐cell contact (Figure 3D). The alignment of fibers appeared to result from cell force‐mediated matrix reorganization and preceded directional extension/migration of cell pairs towards one another by ≈20–30 min (Figure 3D,E; Figure S8A, Supporting Information). Aligned fibers spanning cell pairs were not observed when cells failed to make contact, independent of matrix stiffness, suggesting that cell force‐mediated fiber alignment is a prerequisite to mechanical communication (Figure S8B, Supporting Information).

Two non‐mutually exclusive explanations for a requirement for cell force‐mediated fiber alignment preceding cell‐cell contact include: 1) aligned fibers promote contact guidance cues, enabling directional migration of cell pairs towards each other, and/or 2) aligned fibers enhance force transmission between cells, in turn promoting directional extension/migration via MIC. To ascertain the relative importance of these two scenarios, we patterned EC pairs on pre‐aligned matrices of either low or high stiffness (Figure 3B bottom row, Movie S5, Supporting Information). If contact guidance alone is sufficient for driving cell‐cell contact, we would expect little effect of matrix stiffness on the percent of contacting cells. However, 76.9% of EC pairs (*n* = 26) in cell‐deformable, aligned matrices exhibited cell‐cell interactions, in contrast to 33.3% of EC pairs in stiffer, non‐deformable but aligned matrices (*n* = 24) (Figure 3B,C). Furthermore, in deformable matrices, ECs maintained cell‐cell contact for significantly longer durations compared to in non‐deformable matrices (Figure 3D). Together, these results indicate that force transmission through aligned fibers spanning neighboring cells enhances the transmission of intercellular mechanical signals to facilitate direct cell‐cell contact.

### 2.4 Micropatterned, Multicellular Lines Support a Role for Coordinated Intercellular Ca^2±^ Signaling between Neighboring Cells during MIC

**Figure 4:**
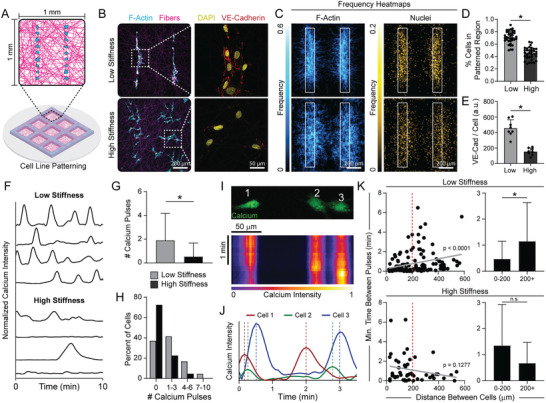
Micropatterned, multicellular lines support a role for coordinated intercellular Ca^2+^ signaling between neighboring cells during MIC. A) Schematic of EC line patterning consisting of two parallel lines of cells within each suspended fibrous matrix. B) Representative confocal fluorescent image of phalloidin‐stained ECs (cyan), rhodamine‐labeled fibers (magenta), nuclei (yellow), and VE‐cadherin (red). Dashed boxes indicate locations of higher magnification images of VE‐cadherin immunostaining (right column). C) F‐actin (left column) and nuclei (right column) heat maps of EC lines 12 h after patterning (*n* = 37 fields of view). D) Quantification of the percent of nuclei in the original patterned region after 12 h (*n* > 35 fields of view). E) Quantification of total VE‐cadherin expression at cell‐cell junctions normalized to cell density for each matrix condition (*n* = 8 fields of view). F) Normalized Ca^2+^ intensity over a 10‐minute time course. G) Quantification of total number of Ca^2+^ pulses per cell and H) distribution of cells by number of Ca^2+^ pulses over a 10‐minute time frame (*n* > 89 cells). I) Representative images of Ca^2+^ signal in ECs within a patterned line with corresponding kymograph and J) normalized Ca^2+^ intensity displaying a wave of Ca^2+^ fluorescence across the line over time. (K) Quantification of the minimum time between Ca^2+^ pulses as a function of distance between all ECs within patterned lines for low (*n* = 160 cell pairs) and high stiffness (*n* = 49 cell pairs) matrix conditions. Grey lines indicate linear correlations with indicated p‐values. All data presented as mean ± SD with superimposed data points; asterisk denotes significance with *p* < 0.05.

We next explored the possibility of harnessing MIC to pattern multicellular assembly into multicellular clusters, mimicking the overall linear cellular organization of capillaries. We further modified our cell/ECM patterning technique to pattern lines of equally spaced cells within a 1 mm^2^ square suspended fiber matrix (**Figure** **4**a). Lines of ECs were patterned on cell‐deformable and non‐deformable matrices to examine how matrix mechanics and resulting MIC regulated the formation and maintenance of organized multicellular structures. ECs patterned in deformable matrices aligned matrix fibers formed stable multicellular structures reflecting the original cell patterning (Figure 4b,c). ECs patterned in non‐deformable matrices, however, appeared to spread and migrate independently, maintaining little fidelity to their original pattern as quantified by the proportion of nuclei within the patterned region after 12 h of culture (Figure 4b–d). Additionally, VE‐cadherin immunostaining to assess the formation of adherens junctions indicated significantly higher VE‐cadherin signal in ECs patterned in deformable matrices as compared to non‐deformable matrices (Figure 4b,e), in agreement with our previous observations of bulk‐seeded matrices (Figure 1g‐i).

Beyond testing the potential to control patterned multicellular assembly, we also employed this culture format to explore how ECs communicate with each other during multicellular assembly. Calcium (Ca^2+^) is critical for many cell functions and in particular, cytosolic Ca^2+^ influx has previously been implicated in mechanosensing due to the presence of stretch activated ion channels present within the plasma membrane of many cell types.^[^ ^45^ ^]^ Increases in intracellular Ca^2+^ have been shown to trigger a wide variety of cellular processes, including cytoskeletal reorganization underlying cell polarization, protrusion formation, and migration.^[^ ^46^, ^47^ ^]^ Thus, we hypothesized that intracellular Ca^2+^ signaling could play an important role in the EC response to cell‐generated mechanical signals in fibrous matrices.

To examine EC Ca^2+^ signaling, cells were patterned into lines on suspended fiber matrices, incubated with a Ca^2+^ sensitive reporter dye, and imaged two hours after patterning (ie. prior to the formation of cell‐cell contacts) to visualize intracellular Ca^2+^ flux as a function of matrix stiffness (Movie S6, Supporting Information). Ca^2+^ activity was significantly higher in ECs in cell‐deformable matrices (Figure 4f), with cells exhibiting more frequent Ca^2+^ signal pulses compared to those in non‐deformable matrices (Figure 4g,h). Additionally, we observed instances of temporally sequenced Ca^2+^ pulses between neighboring cells in deformable matrices, where waves of Ca^2+^ fluxed across the line of assembling ECs (Figure 4i,j). To quantitatively assess the spatiotemporal regulation of Ca^2+^ signaling within EC lines, we determined the minimum time between Ca^2+^ pulses between two ECs as a function of their separation distance, hypothesizing that MIC would facilitate shorter intervals between Ca^2+^ pulses in more proximal cells. Indeed, this correlation was positive and significant in deformable matrices, in stark contrast to non‐deformable matrices (Figure 4k). Interestingly, neighboring ECs within 200 µm from each other (the same distance that interacting cell pairs were patterned in Figure 3) displayed coordinated Ca^2+^ signaling as evidenced by a shorter time interval between Ca^2+^ pulses in deformable matrices (Figure 4k). Neighboring ECs that were further than 200 µm apart, however, had longer average time between peaks with greater variation, indicating a decrease in coordinated Ca^2+^ signaling as a function of distance between cells in deformable matrices. Together, this data indicates that synchronized Ca^2+^ influx underlies MIC between ECs.

### 2.5 Inhibition of Focal Adhesion Kinase Signaling and Mechanosensitive Ion Channels Reduce Ca^2±^ Signaling and MIC between ECs

**Figure 5:**
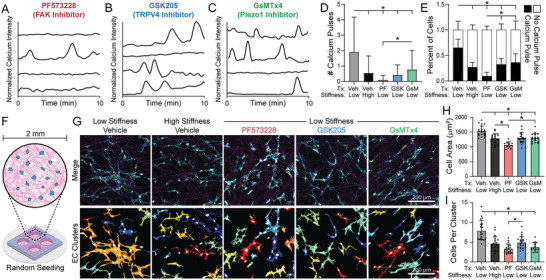
Inhibition of FAK signaling and SAIC activity reduce Ca^2+^ signaling and MIC between ECs. A–C) Normalized Ca^2+^ intensity over a 10‐minute time course of patterned EC lines in low stiffness, cell‐deformable matrices treated with (A) PF573228 (FAK inhibitor), (B) GSK205 (TRPV4 inhibitor) and (C) GsMTx4 (Piezo1 inhibitor). D) Quantification of total number of Ca^2+^ pulses per cell and E) percent of cells with at least one Ca^2+^ pulse over a 10‐minute timeframe (*n* > 84 cells). F) Schematic of EC bulk seeding in 2 mm diameter circular suspended fibrous matrices. G) Confocal fluorescent images of phalloidin‐stained ECs (cyan), nuclei (yellow), and rhodamine‐labeled fibers (magenta) with respective color‐coded maps of contiguous actin clusters as a function of matrix stiffness and presence of inhibitors. H) Quantification of average cell spread area and I) average cells per contiguous actin cluster (*n* > 17 fields of view). All data presented as mean ± SD with superimposed data points; asterisk denotes significance with *p* < 0.05.

After identifying roles for both FAs and Ca^2+^ signaling in MIC, we next inhibited focal adhesion kinase (FAK), transient receptor potential vanilloid 4 (TRPV4), and Piezo1 to examine their respective roles in Ca^2+^ signaling and the formation of multicellular clusters in cell‐deformable fibrous matrices. FAK is a non‐receptor tyrosine kinase that transduces mechanical signals at FAs to intracellular biochemical signals that direct cell signaling and resulting behavior.^[^ ^48^ ^]^ TRPV4 and Piezo1 are stretch‐activated ion channels (SAICs) that open in response to membrane stretch and gait Ca^2+^ influx and regulate intracellular Ca^2+^ signaling in ECs.^[^ ^49^, ^50^ ^]^ Inhibition of FAK with PF573228 (10 µm), TRPV4 with GSK205 (10 µm), or Piezo1 with GsMTx4 (5 µm) all led to a significant decrease in Ca^2+^ pulses compared to ECs in deformable matrices, all to comparable levels to ECs in non‐deformable matrices (**Figure** **5**a–e; Movie S7, Supporting Information). Additionally, inhibition of FAK, TRPV4, or Piezo1 each led to a decrease in spatially coordinated Ca^2+^ signaling but did not affect cell force‐mediated matrix deformations (Figures S9 and S10, Supporting Information). To confirm the role of FAK and SAICs in Ca^2+^ signaling, we treated populations of ECs (250 cells mm^−2^) seeded in a 2 mm diameter suspended fiber matrix (as in our initial studies presented in Figure 1) with the same inhibitors (Figure 5f). Inhibition of FAK, TRPV4, or Piezo1 all led to a decrease in average cell spread area as well as the average number of ECs per cluster after 12 h of culture in deformable matrices, resulting in similar values of these metrics to ECs seeded in high stiffness matrices (Figure 5g–i). These results indicate that FAK signaling and SAIC activity both play an important role in regulating MIC between ECs.

### 2.6 Pharmacologic and Biomaterial Control of MIC Regulates 3D Vascular Network Formation in Fibrin Hydrogels

**Figure 6:**
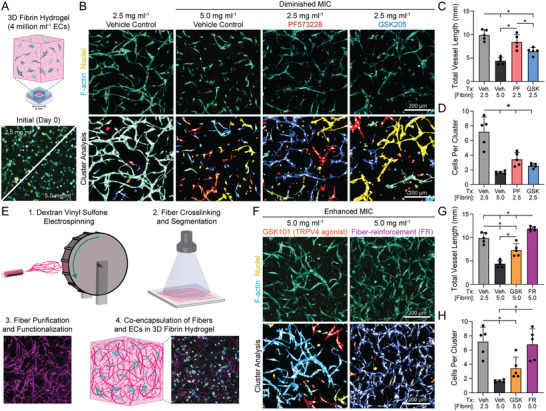
Pharmacologic and biomaterial control of MIC regulates 3D vascular network formation in fibrin hydrogels. (A) Schematic of 3D vascular network formation assay in fibrin hydrogels. (B) Representative confocal maximum intensity projections (100 µm thick z‐stack) of phalloidin‐stained ECs (cyan) and nuclei (yellow) with respective color‐coded maps of contiguous actin clusters as a function of fibrinogen concentration and presence of inhibitors to diminish MIC after 3 days of culture. (C) Quantification of total vessel length and (D) average number of cells in each 3D contiguous actin clusters (*n* = 5 fields of view). (E) Fabrication schematic for generating fiber‐reinforced hydrogels. (F) Representative confocal maximum intensity projections (100 µm thick z‐stack) of phalloidin‐stained ECs (cyan) and nuclei (yellow) with respective color‐coded maps of contiguous actin clusters with cells treated with TRPV4 agonist (GSK101) and addition of DexVS fibers in order to enhance MIC after 3 days of culture. (G) Quantification of total vessel length and (H) average number of cells in each 3D contiguous actin clusters (*n* = 5 fields of view). All data presented as mean ± SD with superimposed data points; asterisk denotes significance with *p* < 0.05.

The 2.5D synthetic fibrous matrices described above provided a highly controllable setting allowing us to investigate how mechanical properties of the ECM regulate MIC between ECs to promote the formation and stabilization of organized multicellular structures. However, harnessing MIC to engineer functional microvascular networks for tissue engineering applications requires the study of EC network formation from populations of ECs embedded within 3D biomaterials. Using 2.5D suspended fibrous matrices, we found that ECs utilize mechanical signaling to communicate to neighboring cells and that this process was highly dependent on matrix fiber stiffness/density, FAK signaling, and SAIC activity. Whether these findings translate to 3D settings that possess translational potential, however, is unclear. As such, we examined vascular network formation in 3D fibrin hydrogels, a commonly used biomaterial platform amenable to vasculogenic assembly, in conditions that should either diminish or enhance MIC between ECs (Figure 6a).^[^ ^51^ ^]^ As a control, ECs (4 million cells mL^−1^) were seeded in 2.5 mg mL^−1^ fibrin hydrogels and cultured for 3 days where they assembled into interconnected networks suggestive of capillary‐scale microvasculature as evidenced by the presence of lumens(**Figure** **6**b; Figure S11, Supporting Information). To diminish MIC mechanically and pharmacologically, we^[^ ^1^ ^]^ increased matrix stiffness/density by increasing fibrinogen concentration to 5.0 mg mL^−1^ or^[^ ^2^ ^]^ inhibited FAK or TRPV4 in control 2.5 mg mL^−1^ fibrin hydrogels, respectively. In all three conditions that putatively diminish MIC, ECs failed to assemble into multicellular networks (Figure 6b–d; Movie S8, Supporting Information), mirroring our findings in 2.5D synthetic fibrous matrices (Figure 5).

As ECs can possess significant molecular and functional differences depending on their tissue origin, we performed comparable studies to examine whether MIC mediates multicellular assembly of human lung microvascular endothelial cells (HLMVECs).^[^ ^52^, ^53^, ^54^ ^]^ Interestingly, HLMVECs were capable of vasculogenic assembly at higher fibrin densities (5.0 mg mL^−1^), which proved inconducive to vasculogenic assembly of HUVECs (Figure 6; Figure S12a, Supporting Information). This difference may be attributed to enhanced angiogenic potential of microvascular cells as compared to HUVECs.^[^ ^54^, ^55^ ^]^ HLMVECs formed vascular assembly in 5.0 mg mL^−1^ fibrin hydrogels, but demonstrated diminished capacity to assemble at higher fibrin densities (7.5 and 10 mg mL^−1^), in line with the anticipated decrease in MIC with increasing matrix density (Figure S12a, Supporting Information). Additionally, similar to our findings with HUVECs, HLMVECs treated with PF228 (FAK inhibitor) or GSK 205 (TRPV4 inhibitor) both resulted in reduced networks (Figure S12e,f, Supporting Information).

Lastly, we tested whether enhancing MIC could promote vasculogenic assembly in 3D fibrin hydrogels at densities that proved otherwise non‐permissive. To do so via a biomaterials‐based strategy, we utilized a previously established methodology for generating fiber‐reinforced hydrogel composites.^[^ ^56^ ^]^ Briefly, dextran vinyl sulfone (DexVS) fiber segments were electrospun, photopatterned into defined lengths, collected in solution, and functionalized with the cell‐adhesive peptide RGD. These cell‐adhesive fibers were then incorporated into fibrin hydrogels alongside ECs (Figure 6e). Indeed, the addition of synthetic matrix fibers (which are larger in diameter and persistence length than fibrin fibrils) promoted network assembly compared to non‐reinforced fibrin hydrogels of the same density (Figure 6f–h). Similar results were observed when HLMVECs were cultured in fiber‐reinforced fibrin hydrogels at a fibrin density that otherwise precludes network assembly (Figure S12b, Supporting Information). Using pharmacologic means to enhance MIC, addition of the TRPV4 agonist GSK101 (100 nm) in control 2.5 mg mL^−1^ fibrin hydrogels also led to enhanced HUVEC network formation in 3D (Figure 6f–h). These results support our findings in synthetic fibrous matrices and support a critical role for MIC during 3D vascular network formation mediated by FAK signaling and TRPV4 activity.

## 3 Discussion

MIC involves the generation, transmission, and receipt of cell‐generated forces conveyed through the ECM, which we posit to be an important but understudied means of intercellular communication. Here, we utilized synthetic fibrous matrices with tunable mechanics in conjunction with a microfabrication‐based cell patterning approach to better understand how physical properties of the matrix modulate MIC between ECs. We first varied cell seeding density (and resulting distance between adhered cells) in low and high stiffness fibrous matrices and found that soft, cell‐deformable ECM fibers supported heightened cell‐ECM adhesion that corresponded with enhanced cell spreading, migration, and the eventual formation of multicellular clusters (Figure 1). Cell/ECM patterning experiments with either single cells or cell pairs revealed that ECs in soft, cell‐deformable matrices favored fewer but longer lasting protrusions, generated longer‐range matrix displacements, and enabled directional migration towards neighboring cells that preceded the formation of stable cell‐cell connections via VE‐cadherin (Figures 2 and 3). We also utilized this method to explore the cellular machinery responsible for MIC, finding a critical role for intracellular Ca^2+^ signaling mediated by FAK, TRPV4, and Piezo1 during the assembly of multicellular clusters in deformable matrices (Figures 4 and 5). Finally, we tested whether our observations of MIC in synthetic fiber matrices could be extrapolated to more translatable 3D settings by examining EC network assembly in fibrin hydrogels. Indeed, biomaterial or pharmacological manipulation to diminish or enhance MIC led to abrogation and enhancement of EC network assembly in 3D matrices, respectively (Figure 6).

Fibrous matrices have been theorized to promote long‐range matrix displacements in a variety of computational models; these studies implicate a role for fiber alignment, strain stiffening, and ECM fiber microbuckling in propagating cell‐generated forces.^[^ ^25^, ^26^, ^27^, ^28^, ^29^, ^30^, ^31^, ^32^, ^33^, ^34^ ^]^ However, exploring cell‐force generation and propagation in fibrous matrices in vitro is experimentally challenging due the discrete (non‐affine), non‐linear, and plastic mechanical behavior of most naturally derived fibrous ECMs. Here, we utilized mechanically tunable synthetic fibrous matrices along with timelapse imaging to quantify matrix deformations and found that low stiffness fibrous matrices support longer‐range matrix displacements. Additionally, our analyses of cell morphology and cell‐ECM adhesion suggest that deformable fibrous matrices prime cells for enhanced and directed force generation and transmission. In experiments with single ECs patterned in deformable matrices, cells exhibited increased FA size and decreased number of protrusions compared to ECs patterned in stiff, non‐deformable matrices, despite encountering the same initial ligand density and matrix topography. This divergence may be explained by matrix stiffness‐dependent differences in mechanical resistance during cell spreading. In high stiffness matrices, all matrix fibers provide sufficient mechanical resistance required for FA assembly and associated protrusion formation, thereby prompting cells to isotropically extend in a random fashion. In contrast, ECs in low stiffness matrices rapidly recruit matrix fibers soon after initial adhesion and generate tension in a subset of fibers depending on their connection to a local rigid boundary (in this case, the edge of the microfabricated well). This likely results in greater heterogeneity of mechanical resistance, leading to preferential generation of FAs and protrusions along particular fibers that provide heightened mechanical resistance, resulting in more directional extension and force propagation. Future work could combine our cell/ECM patterning approach with intramolecular or polymeric tension sensors to better understand the relationship between forces at FAs, protrusive activity, and directional migration.^[^ ^57^ ^]^

Beyond influencing cell‐ECM adhesion and spreading, matrix deformations and reorganization resulting from cell traction forces in low stiffness matrices also led to matrix structural and mechanical anisotropy via local fiber alignment. When cell pairs were patterned in deformable, randomly oriented fibrous matrices, we frequently observed fiber alignment between neighboring cells preceding directional extension, migration, and formation of cell‐cell contact. Several possible explanations exist. Fiber alignment could provide a topographical cue that guides directed migration between cells via contact guidance.^[^ ^58^ ^]^ Additionally, and non‐exclusively, aligned fibers spanning cell pairs could maximize the transmission of tensile forces between cells and thereby enhance MIC. Our results suggest active force transmission across aligned fibers enhances MIC, as high stiffness aligned matrices that cannot deform under cell forces led to fewer cell‐cell interactions compared to softer matrices with the same aligned topography (Figure 5). In recent work, Pakshir et al. observed that contracting myofibroblasts generate large deformation fields in collagen matrices that provide mechanical signals to macrophages.^[^ ^59^ ^]^ However, it was also observed that cell force‐mediated fiber alignment from myofibroblasts was not required to guide macrophage migration. These conflicting observations may arise from distinct cell types or different ECM settings, as reconstituted collagen hydrogels have relatively short fibrils with nanometer‐scale diameters compared to longer electrospun DexMA fibers with larger diameters used in these studies.^[^ ^36^ ^]^ The difference in cell‐ECM interaction due to differences in cell types was also observed in our study, where HLMVECs were able to form vascular networks in higher fibrin densities (5.0 mg mL^−1^) considered inconducive for vasculogenic assembly for HUVECs. This heightened ability of MVECs to assemble networks is evidenced by their ability to proliferate and support capillary outgrowth from cell spheroids in collagen gels without the supplementation of angiogenic growth factors.^[^ ^55^ ^]^ On the contrary, in the absence of exogenous growth factors HUVECs had limited proliferation and capillary outgrowth.^[^ ^55^ ^]^ Similarly, a different study demonstrated that microvascular ECs express angiogenic genes to a higher extent than macrovascualr cells such as HUVECs, with these genes having been shown to modulate cytoskeletal remodeling and migration in angiogenesis.^[^ ^54^ ^]^ Taken together, however, our results confirm that various cell types are able to generate and respond to dynamic mechanical signals in fibrous matrices.

While evidence for MIC has been observed in a variety of settings, the machinery required for ECs to send, receive, and respond to mechanical signals during network assembly has not been established. Here, we identified critical roles for FAK signaling and SAICs in EC MIC. Inhibition of FAK significantly decreased Ca^2+^ signaling and multicellular assembly in matrices permissive to MIC (Figures 5 and 6; Figure S12e,f, Supporting Information). While FAK inhibition has not been shown to influence FA maturation or traction force magnitudes, it does play an important role in adhesion dynamics and cell motility.^[^ ^60^, ^61^, ^62^ ^]^ Plotnikov et al. investigated FA traction dynamics in mouse embryonic fibroblasts and found FAK activity was required for “tugging‐like” traction force fluctuations from FAs.^[^ ^62^ ^]^ This could in part explain why FAK inhibition abrogated Ca^2+^ fluxes in soft matrices, as we expect that dynamic traction forces transmitted through the ECM are critical for MIC.^[^ ^59^ ^]^ Furthermore, cells likely sense dynamic mechanical signals from the ECM via membrane stretch that triggers the opening of SAICs. Here, we identified a critical role for both Piezo1 and TRPV4, two SAICs that are expressed in ECs and are known to be important during the EC response to shear stress in other contexts.^[^ ^49^, ^50^ ^]^ Beyond regulating EC response to shear stress, Thodeti et al. showed that TRPV4 mediates EC reorientation under cyclic mechanical stretch.^[^ ^49^ ^]^ In their experiments, uniaxial cyclic strain of flexible substrates seeded with ECs led to cellular realignment perpendicular to the axis of applied strain in a TRPV4 dependent manner. The authors posit that ECs preferentially align perpendicular to the direction of applied strain due to differences in membrane strain, TRPV4 activation, and resulting cytoskeletal activity parallel versus perpendicular to the direction of stretch. This could also explain alignment of cells and directional protrusions in response to cell‐generated forces during MIC, as regions of the plasma membrane proximal to a force‐generating neighboring cell are expected to experience higher levels of stretch than more distal regions. Direct measurements of membrane tension and ion channel state could provide more information on the spatial sensitivity of MIC.

A deeper understanding of vasculogenic assembly could inform the design of biomaterials that better facilitate the self‐assembly of functional vascular networks in vitro for tissue engineering and regenerative medicine applications. Our studies show that matrices that permit cellular force transmission via long‐range matrix deformations enable MIC, and furthermore that MIC is required for the assembly and stabilization of microvascular structures. Interestingly, vasculogenic assembly is readily achieved in collagen and fibrin hydrogels – two materials where evidence of MIC has been well‐documented.^[^ ^51^, ^59^, ^63^, ^64^ ^]^ These materials, however, hold limited potential for building engineered tissue constructs due to their rapid resorption in vivo.^[^ ^65^ ^]^ Alternatively, synthetic polymeric hydrogels, such as poly(ethylene) glycol, hyaluronic acid, and dextran, offer controllable and modular design better suited for translational applications.^[^ ^66^ ^]^ However, compared to the aforementioned natural and fibrous materials, building 3D vascular networks has proven more challenging in these synthetic biomaterial settings. Based on our studies, one explanation for the challenges of assembling vascular networks in 3D synthetic hydrogels may be the dissipation of cell forces short distances away from the cell given the non‐fibrous architecture of these materials.^[^ ^67^, ^68^ ^]^ Generally, EC spreading and network formation in these settings requires the addition of a support stromal cell such as dermal fibroblasts, mesenchymal stem cells, or pericytes. The role of these stromal cells has long been considered biochemical in nature via growth factor and matrix secretion or ECM degradation.^[^ ^69^, ^70^, ^71^ ^]^ However, in addition to biochemical support, these contractile cells may also provide mechanical signals that orchestrate EC network formation. Supporting this notion, recent work from Song et al. found that fibroblasts present during 3D EC network formation in fibrin are only necessary during the first few days of culture during assembly, after which they can be selectively ablated without long‐term negative effects on formed vascular networks.^[^ ^72^ ^]^

Additionally, external mechanical cues applied by actuated or dynamic biomaterials could guide cell extension and migration to form complex multicellular patterns,^[^ ^73^ ^]^ (q‐s). Beyond playing an important role during the bottom‐up assembly of multicellular vascular structures, our results indicate a role for MIC and force transmission in the stabilization and maturation of stable, multicellular EC structures, evidenced here by enhanced junctional VE‐cadherin localization in EC clusters and patterned EC cords formed in cell‐deformable matrices. The influence of matrix mechanics and MIC on the assembly and stability of patterned cells should be considered in top‐down approaches to vascular tissue engineering, such as 3D bioprinting of ECs.^[^ ^74^ ^]^ While bioprinting techniques continually improve in resolution and complexity and now allow for cell‐scale patterning, an understanding of how matrix properties influence the formation and maintenance of cell‐cell adhesions is critical. As such, biomaterials optimized for vasculogenic assembly should consider not only initial phases of assembly, but also the long‐term maintenance and function of multicellular structures.

## 4 Experimental Section

### Reagents

All reagents were purchased from Sigma‐Aldrich and used as received, unless otherwise stated.

### Cell Culture and Biological Reagents

Human umbilical vein endothelial cells (ECs) were cultured in endothelial growth medium (EGM‐2; Lonza, Basel, Switzerland) supplemented with 1% penicillin‐streptomycin‐fungizone (Gibco, Waltham, MA). Human lung microvascular endothelial cells (HLMVECs) were cultured in endothelial growth medium (EGM‐2) supplemented with extra fetal bovine serum (FBS; 3% v/v), and 1% penicillin‐streptomycin‐fungizone. Cells were cultured at 37 °C and 5% CO_2_. HUVECs and HLMVECs were used from passages two to six and four to eight respectively in all experiments. For live cell time‐lapse imaging, lentiviral transduction of LifeAct‐GFP was utilized. For inhibition studies, PF‐573228 (10 µm), GSK205 (10 µm; Medchem Express, Monmouth Junction, NJ), and GsMTx‐4 (5 µm; Abcam, Cambridge, UK) were supplemented in EGM‐2 and refreshed every 24 h.

### Lentivirus Production

pLenti.PGK.LifeAct‐GFP.W was a gift from Rusty Lansford (Addgene plasmid #51 010). To generate lentivirus, plasmids were co‐transfected with pCMV‐VSVG (a gift from Bob Weinberg, Addgene plasmid #8454), pMDLg/pRRE, and pRSV‐REV (gifts from Didier Trono, Addgene plasmid #12 251 and #12 253^[^ ^75^, ^76^ ^]^) in 293T cells using the calcium phosphate precipitation method.^[^ ^77^ ^]^ Viral supernatants were collected after 48 h, concentrated with PEG‐it (System Biosciences, Palo Alto, CA) following the manufacturer's protocol, filtered through a 0.45 µm filter (ThermoFisher Scinetific Nalgene, Waltham, MA), and stored at −80 °C. Viral titer was determined by serial dilution and infection of ECs. Titers yielding maximal expression without cell death or detectable impact on cell proliferation or morphology were selected for studies.

### DexMA Synthesis

Dextran (MW 86 000 Da, MP Biomedicals, Santa Ana, CA) was methacrylated by reaction with glycidyl methacrylate as previously described.^[^ ^78^ ^]^ Briefly, 20 mg of dextran and 2 mg of 4‐dimethylaminopyridine were dissolved in 100 mL of anhydrous dimethylsulfoxide (DMSO) under vigorous stirring (300 rpm) for 12 h. 24.6 mL of glycidyl methacrylate was then added and the reaction mixture was heated to 45 °C for 24 h. The solution was cooled at 4 °C for 1 h and precipitated into 1 L ice‐cold 2‐isopropanol. The crude product was recovered by centrifugation, redissolved in milli‐Q water, and dialyzed against milli‐Q water for 3 d. The final product was lyophilized and stored at −20 °C until use. DexMA was characterized by ^1^H‐NMR. The degree of functionalization was calculated as the ratio of the averaged methacrylate proton integral (6. 174 ppm and 5.713 ppm in D2O) and the anomeric proton of the glycopyranosyl ring (5.166 ppm and 4.923 ppm). As the signal of the anomeric proton of α−1,3 linkages (5.166 ppm) partially overlaps with other protons, a pre‐determined ratio of 4% α−1,3 linkages was assumed and the total anomeric proton integral was calculated solely on the basis of the integral at 4.923 ppm. A methacrylate/dextran repeat unit ratio of 0.787 was determined.

### Fiber Matrix Fabrication

Suspended DexMA fiber matrices were fabricated through electrospinning and soft lithography as previously described.^[^ ^35^ ^]^ DexMA was dissolved at 0.5 g mL^−1^ in a 1:1 mixture of milli‐Q water and dimethylformamide with 1% (w/v) Irgacure 2959 photocrosslinker and 0.625 mm methacrylated rhodamine (Polysciences, Inc., Warrington, PA). For matrix displacement studies, 10% (v/v) blue carboxylate‐modified FluoSpheres (1.0 µm diameter, 2% w/v) was also added. Electrospinning was completed with a custom set‐up consisting of a high‐voltage power supply (Gamma High Voltage Research, Ormond Beach, FL), syringe pump (KD Scientific, Holliston, MA), and a grounded copper collecting surface enclosed within an environmental chamber held at room temperature and 30% relative humidity (Terra Universal, Fullerton, CA). Electrospinning of DexMA solution was performed at a flow rate of 0.45 mL h^−1^, voltage of 7.0 kV, and gap distance of 6 cm. Fiber density was varied through modulating electrospinning time and relative humidity. To induce fiber alignment, fibers were electrospun at a voltage of 4.0 kV onto a collecting surface of oppositely charged (−3.0 kV) parallel electrodes at a 25 mm separation distance. After electrospinning, fibers were stabilized by primary crosslinking under ultraviolet (UV) light (100 mW cm^−2^) for 60 s, hydrated in varying concentrations of lithium phenyl‐2,4,6‐trimethylbenzoylphophinate (LAP; Colorado Photopolymer Solutions, Boulder, CO) photoinitiator solution, and then exposed again to UV light (100 mW cm^−2^) for 20 s. Low, intermediate, and high stiffness networks were crosslinked in 0.02, 0.075, and 1.0 mg mL^−1^ LAP solutions, respectively. Fibers were collected on various poly(dimethylsiloxane) (PDMS; Dow Silicones Corporation, Midland, MI) arrays of wells produced by soft lithography. Silicon wafer masters possessing SU‐8 photoresist (Microchem, Westborough, MA) were first fabricated by standard photolithography. Briefly, a layer of SU‐8 2075 (110 µm thick) was spin‐coated on a 3‐inch silicon wafer and patterned into arrays of various shaped wells spaced evenly within 12×12 mm squares. These masters were utilized to make PDMS stamps which were silanized with trichloro(1H,1H,2H,2H‐perfluorooctyl)silane and used to emboss uncured PDMS onto oxygen plasma‐treated coverslips. Resultant fiber‐well substrates were methacrylated by vapor‐phase silanization of 3‐(trimethoxysilyl)propyl methacrylate in a vacuum oven at 60 °C for at least 6 h to promote fiber adhesion to PDMS.

### Mechanical Testing

To determine the Young's modulus of suspended DexMA fibrous matrices, microindentation testing with a rigid cylinder was performed on a commercial CellScale Microsquisher (CellScale, Waterloo, Ontario). Briefly, samples were indented to a depth of up to 200 µm at an indentation speed of 2 µm s^−1^, and Young's modulus was approximated assuming the material behaves as an elastic membrane as previously described.^[^ ^35^ ^]^

### RGD Functionalization and Seeding on DexMA Matrices

DexMA fibers were functionalized with the cell adhesive peptide CGRGDS (RGD; Peptides International, Louisville, KY). An RGD concentration of 2 mm was used for all studies. RGD was coupled to available methacrylates via Michael‐type addition. Briefly, the peptide was dissolved in milli‐Q water containing HEPES (50 mm), phenol red (10 µg mL^−1^), and 1 M NaOH to adjust the pH to 8.0. 250 µL of this solution was added to each substrate and incubated for 30 min at room temperature. Following RGD functionalization, substrates were rinsed 2x with PBS before cell seeding. For bulk seeding of networks, ECs were trypsinized, resuspended in 1.5% (w/v) methylcellulose supplemented EGM‐2 to increase media viscosity, and seeded between 50 and 250 cells mm^−2^.

### Fluorescent Staining and Microscopy

ECs on DexMA fibers were first fixed in 4% paraformaldehyde for 10 min at room temperature. Alternatively, to extract cytoplasmic vinculin, samples were simultaneously fixed and permeabilized in 2% paraformaldehyde in a microtubule‐stabilizing buffer containing 1,4‐piperazinediethanesulfonic acid (PIPES, 0.1 m), ethylene glycol‐bis(2‐aminoethylether)‐N,N,N’,N’‐tetraacetic acid (EGTA, 1 mm), magnesium sulfate (1 mm), poly(ethylene glycol) (4% w/v), and triton X‐100 (1% v/v) for 10 min at room temperature. To stabilize the fibers for processing and long‐term storage, DexMA samples were crosslinked in 2 mL LAP solution (1 mg mL^−1^) and exposed to UV light (100 mW cm^−2^) for 30 s. To stain the actin cytoskeleton and nuclei, cells were permeabilized in PBS solution containing triton X‐100 (5% v/v), sucrose (10% w/v), and magnesium chloride (0.6% w/v), and simultaneously blocked in 1% (w/v) bovine serum albumin and stained with phalloidin and DAPI. For immunostaining, samples were blocked for 1 h in 1% (w/v) bovine serum albumin and incubated with mouse monoclonal anti‐vinculin antibody (1:1000, Sigma #V9264) or mouse monoclonal anti‐VE‐cadherin antibody (1:1000, Santa Cruz #sc‐9989) followed by secondary antibody (1:1000, Life Technologies #A21236) for 1 h each at room temperature with 3x PBS washes in between. Fixed samples were imaged on a Zeiss LSM800 laser scanning confocal microscope. Unless otherwise specified, images are presented as maximum intensity projections. Fluorescent images were processed and quantified via custom Matlab scripts.

### Cell Migration Analysis

Immediately after seeding, substrates were transferred to a motorized and environmentally controlled stage and imaged using a Zeiss LSM800 laser scanning confocal microscope (Zeiss, Oberkochen, Germany). Prior to imaging, cell nuclei were labeled with Hoechst 33 342 (5 µg mL^−1^) for 10 min. F‐Actin, DexMA fibers, and Hoechst‐labeled nuclei were imaged at 10 min frame intervals over 12 h. Following raw image export, images were converted to maximum intensity projections, and cell spreading and cluster formation were quantified using a custom Matlab script. Nuclei tracking was completed with TrackMate, a freely available ImageJ plugin.^[^ ^79^ ^]^ Nuclei were detected at each time point using a Laplacian of Gaussian (LoG) detector with an estimated particle diameter of 20 µm and threshold of 0.01 with use of a median filter. Single particle tracking was completed using a linear assignment problem tracker with a linking max distance and gap‐closing distance of 20 µm and gap‐closing max frame gap of 2 frames. Tracks were filtered to only contain nuclei detected through the entire time‐lapse, and migration speed for each cell was calculated via custom Matlab scripts.

### Microwell Patterning Stamp Fabrication

To pattern single ECs onto suspended matrices of DexMA fibers, we designed a patterning system inspired by a previously developed microwell‐based approach.^[^ ^44^ ^]^ Like the fiber‐well substrates, microwell patterning stamps were produced by soft lithography. First, silicon wafer masters were fabricated with two steps of photolithography. First, SU‐8 2075 (100‐200 µm thick) was spin‐coated on a silicon wafer and patterned into 12.05×12.05 mm elevated squares. This step allowed for the final patterning stamp to be aligned to the square fiber‐well substrate (12×12 mm) during patterning. Next, SU‐8 2025 (35 µm thick) was spin‐coated on top of the previous exposed layer and patterned into arrays of micro‐posts (30 µm diameter) centered onto each 12.05 mm square. Non‐exposed SU‐8 was washed off through the developing process and these masters were utilized to make PDMS microwell patterning stamps as described above.

### Cell Patterning on DexMA Matrices

Microwell patterning stamps were sterilized with 70% ethanol and UVO (Jelight Company Inc., Irvine, CA) for 5 min followed by treatment with 0.2% Pluronic F127 to prevent cell adhesion. 500 µL of EC suspension (1 × 10^6^ cells mL^−1^) was seeded on the patterning stamp and the cells were allowed to settle into the microwells for 5 min. The supernatant was subsequently removed and excess, untrapped cells were gently flushed away with 4 rinses of PBS and 1 rinse of EGM‐2. The fiber‐well substrate containing RGD‐functionalized DexMA fiber matrices was carefully inverted on top of the patterning stamp and aligned. This assembly was then inverted to allow single ECs trapped in microwells to settle and adhere to the DexMA fiber matrix for 15 min (Figure S2, Supporting Information). The final substrate containing patterned ECs on DexMA matrices was then hydrated with EGM‐2 supplemented with HEPES (25 mm) to regulate media pH and minimize hydrolysis‐mediated fiber degradation during culture.

### Timelapse Fiber Displacement Microscopy

Immediately after patterning, substrates were transferred to a motorized and environmentally controlled stage and imaged using a Zeiss LSM800 laser scanning confocal microscope. Images of F‐actin, DexMA fibers, and fluorescent microspheres embedded in fibers were acquired every 10 min for 12 h. Images were converted to maximum intensity projections before analysis. Single particle tracking was completed by first aligning image stacks of fluorescent microspheres, cropping the images to only track microspheres within fibers of the suspended matrix, and tracked using TrackMate. Microspheres were detected at each time point using a Laplacian of Gaussian (LoG) detector with an estimated particle diameter of 5 µm and threshold of 1.0 with use of a median filter. Single particle tracking was completed using a linear assignment problem tracker with a linking max distance and gap‐closing max distance of 20 µm and gap‐closing max frame gap of 2 frames. Tracks were filtered to only contain particles detected throughout the entire time‐lapse and then analyzed using custom Matlab scripts. Additionally, cell morphology, migration, and protrusions were analyzed using custom Matlab scripts.

### Calcium Imaging

Ca^2+^ handling analysis was performed by incubating cells for 1 h at 37 °C with 5 µm Cal520, AM (AAT Bioquest, Sunnyvale, CA) in EGM‐2. Cells were then returned to normal EGM‐2 and allowed to equilibrate for 30 min. Following equilibration, substrates were transferred to a motorized and environmentally controlled stage and imaged using a Zeiss LSM800 laser scanning confocal microscope. Timelapse movies of Ca^2+^ flux were analyzed with custom Matlab scripts.^[^ ^80^ ^]^

### 4.1 Vascular Network Formation

ECs were encapsulated (4 million mL^−1^) in fibrin precursor solutions containing varying concentration of fibrinogen [2.5 mg mL^−1^, 5.0 mg mL^−1^, 7.5 mg mL^−1^, 10 mg mL^−1^, and 15 mg mL^−1^] from bovine plasma and 1 U mL^−1^ bovine thrombin. This precursor solution was mixed and 25 µL was transferred into a PDMS mold with 4 mm diameter and incubated at 37 °C for 20 min. Samples were then hydrated in EGM‐2 supplemented with fetal bovine serum (FBS; 5% v/v), vascular endothelial growth factor (VEGF; 50 ng mL^−1^), and phorbol 12‐myristate 13‐acetate (PMA; 25 ng mL^−1^), and media was replaced every 24 h. Quantification of morphological network properties was performed on 100 µm image stacks. Total vessel length was quantified using AngioTool,^[^ ^81^ ^]^ and 3D analysis of the number cells per contiguous actin structure was completed using a custom Matlab script.

### Statistical Analysis

All experiments were conducted across three or more independent biologic replicates, each performed with a unique set of cells. The number of cells, cell pairs, or fields of view indicated in each figure legends represents the minimum number across all experimental conditions for the presented representative study. Statistical significance was determined by one‐way or two‐way analysis of variance (ANOVA) with post‐hoc analysis (Tukey test) or Student's *t*‐test where appropriate. Additionally, statistical significance of the proportions of interacting and non‐interacting cells was determined by Fisher's exact test. For all studies, significance was indicated by *p* < 0.05. Sample size is indicated within corresponding figure legends and all data are presented as mean ± standard deviation.

## Conflict of Interest

The authors declare no conflict of interest.

## Author Contributions

C.D.D. and F.S.M. contributed equally to this work. C.D.D., F.S.M., and B.M.B. performed conceptualization. C.D.D., F.S.M., S.J.D.P., J.L.K., W.Y.W., D.K.P.J., M.E.W. performed data acquisition. C.D.D., F.S.M., and B.M.B. performed data analysis and interpretation. C.D.D., F.S.M., and B.M.B. performed writing. All authors have read and approved the final submitted manuscript.

## Supporting information

Supporting Information

Supplemental Movie 1

Supplemental Movie 2

Supplemental Movie 3

Supplemental Movie 4

Supplemental Movie 5

Supplemental Movie 6

Supplemental Movie 7

Supplemental Movie 8

## Data Availability

The data that support the findings of this study are available from the corresponding author upon reasonable request.
