# Supplementary material for: Mechanical Intercellular Communication via Matrix‐Borne Cell Force Transmission During Vascular Network Formation
Source: Adv Sci (Weinh). 2023 Nov 23;11(3):2306210. doi: 10.1002/advs.202306210 (PMC10797481; doi:10.1002/advs.202306210)
Supplement: Supplementary file 1 — Supporting Information [file ADVS-11-2306210-s007.pdf]

ADVANCED SCIENCE
Open Access

Supporting Information

for *Adv. Sci.*, DOI 10.1002/advs.202306210

Mechanical Intercellular Communication via Matrix-Borne Cell Force Transmission During Vascular Network Formation

*Christopher D. Davidson, Firaol S. Midekssa, Samuel J. DePalma, Jordan L. Kamen, William Y. Wang, Danica Kristen P. Jayco, Megan E. Wieger and Brendon M. Baker**

Supplemental Information

Mechanical intercellular communication via matrix-borne cell force transmission during vascular network formation

Christopher D. Davidson[1*], Firaol S. Midekssa[1*], Samuel J. DePalma[1], Jordan L. Kamen[1], William Y. Wang[1], Danica Kristen P. Jayco[1], Megan E. Wieger[1], Brendon M. Baker[1,2,+]

[1] Department of Biomedical Engineering, University of Michigan, Ann Arbor, MI 48109

[2] Department of Chemical Engineering, University of Michigan, Ann Arbor, MI 48109

* Authors contributed equally to this work.

[+] Corresponding Author:

Brendon M. Baker, Ph.D.

Associate Professor, Department of Biomedical Engineering, University of Michigan

2174 Lurie BME Building, 1101 Beal Avenue

Ann Arbor, MI 48109

Email: bambren@umich.edu

SUPPLEMENTAL FIGURES

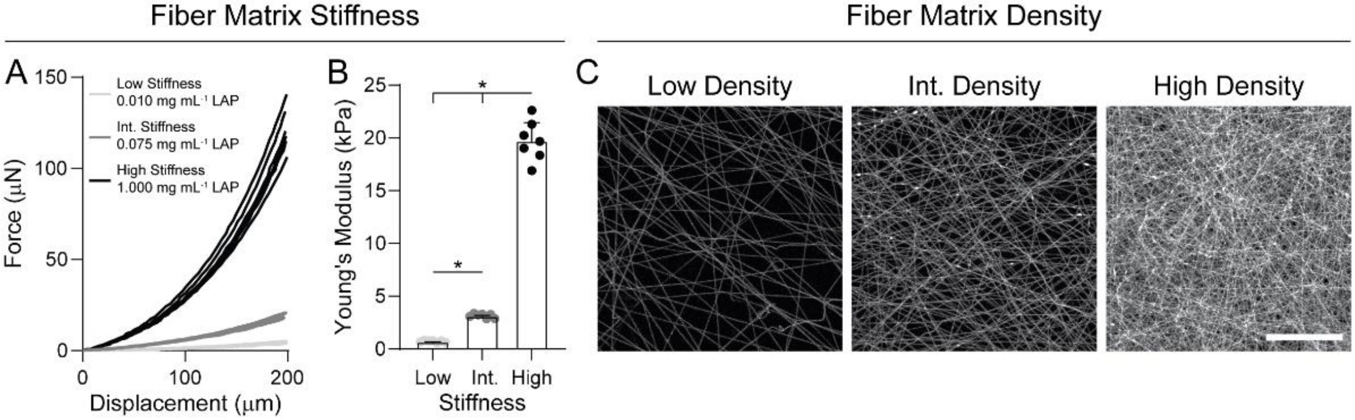

Figure S1. Control over substrate stiffness and fiber density in DexMA fiber networks. (A) Force-indentation response and (B) Young's modulus of DexMA fibrous matrices as a function LAP photoinitiator concentration (n = 7 matrices/group). (C) Representative fluorescent images of DexMA fibers at low, intermediate, and high fiber density (scale bar, 100 μm).

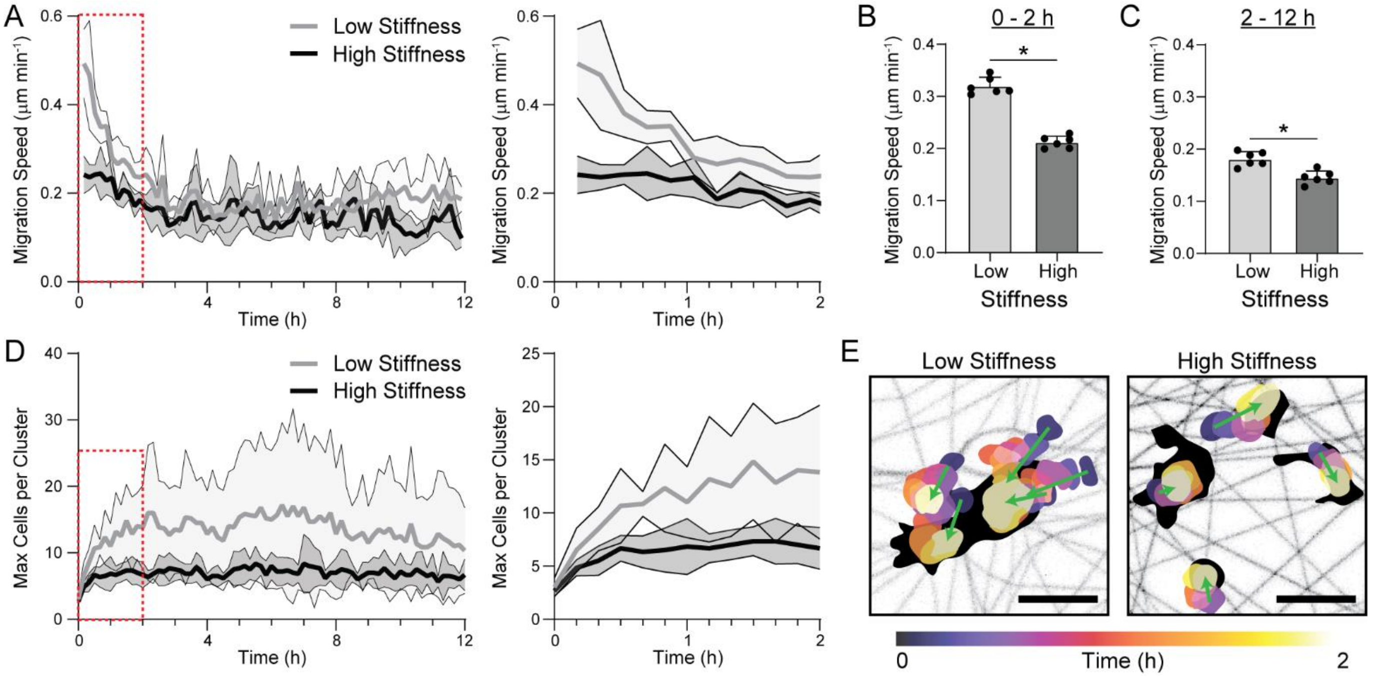

Figure S2. Increase cell migration speeds occur in low stiffness, cell-deformable fibrous matrices prior to the formation of multicellular clusters. (A) Migration speed of ECs over 12 hours following seeding in either low stiffness, cell-deformable matrices or high stiffness, non-deformable matrices (n = 6 fields of view). (B) Quantification of migration speed during the first 2 hours and (C) remaining 10 hours of culture as a function of matrix stiffness (n = 6 fields of view). (D) Maximum cluster size over 12 hours of culture as a function of matrix stiffness (n = 6 fields of view). (E) Temporally color-coded overlay capturing the motion of nuclei over a 2-hour time course in low and high stiffness matrices. Green arrows represent direction of movement for each individual nuclei with eventual contiguous F-actin structures demarcated in black (scale bars, 50 μm). All data presented as mean ± SD with superimposed data points; asterisk denotes significance with $P < 0.05$.

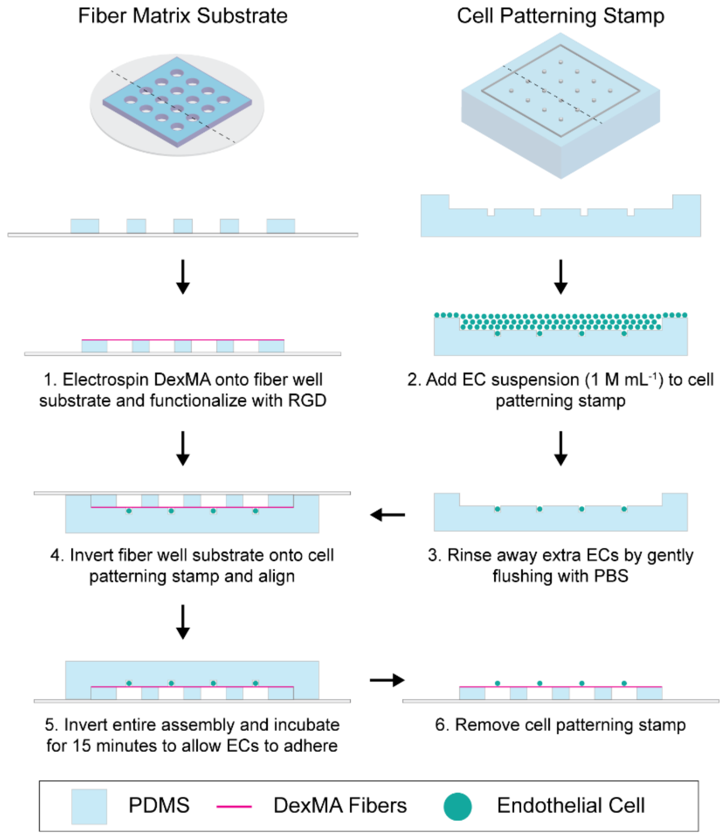

Figure S3. Schematic of endothelial cell micropatterning on suspended DexMA fiber matrices. (A) Isometric and side views of fiber matrix substrate and cell patterning stamp. (B) (1) DexMA fibers are electrospun onto a fiber well substrate, crosslinked, and hydrated. (2) The cell patterning stamp is treated with Pluronic F127 solution to prevent cell adhesion, and a cell suspension is seeded onto the stamp. Cells are allowed to settle for 5 minutes. (3) Excess cells are gently flushed away with 4x PBS rinses. (4) The arrayed fiber well substrate is inverted onto the cell patterning stamp and aligned using the raised edges of the patterning stamp under a microscope. (5) The entire assembly is inverted to allow cells to settle onto the suspended DexMA matrices and incubated for 15 minutes to allow for cell attachment to fibers. (6) The patterning stamp is removed and the cell-patterned matrices are subsequent cultured or imaged by time-lapse microscopy.

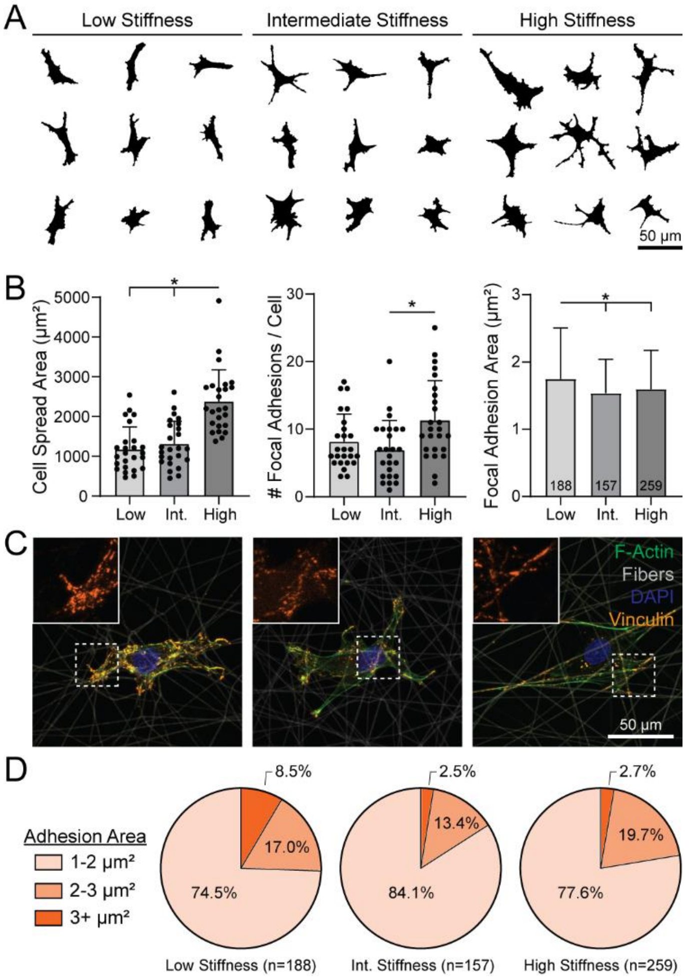

Figure S4: Micropatterning single ECs on suspended fibrous matrices reveals cell spreading and FA formation are matrix stiffness-dependent. (A) Cell outlines of nine representative cells as a function of matrix stiffness. (D) Representative anti-vinculin immunostained images of FAs in ECs as a function of matrix stiffness; F-actin (green), DexMA fibers (grey), nuclei (blue), and vinculin (orange). (E) Quantification of cell spread area (n = 25 cells), (F) number of FAs per cell (n = 25 cells), and (G) average FA area as a function of matrix stiffness. Data presented as mean ± SD with superimposed data points; asterisk denotes significance with $p < 0.05$. (H) Distribution of individual FA area as a function of matrix stiffness showing a larger population of 3+ μm^2 FAs in low stiffness matrices.

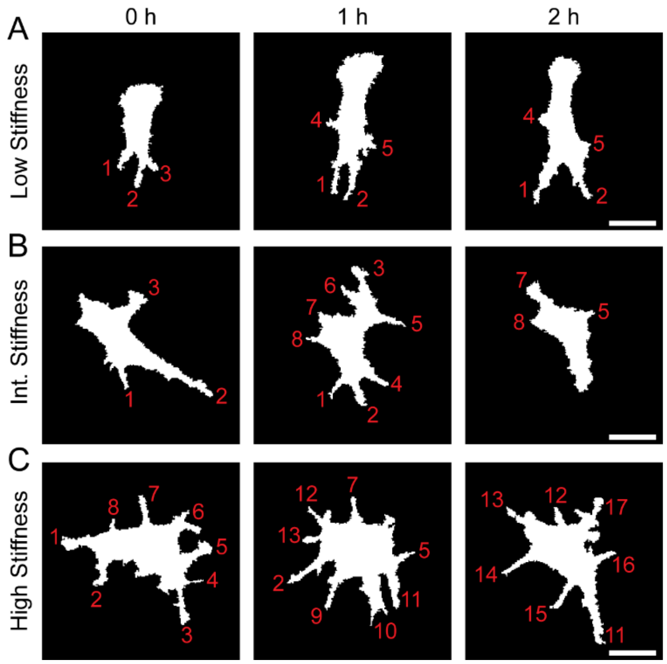

Figure S5. Endothelial cell protrusion analysis. For each frame of the 12-hour time-lapse dataset, protrusions were manually identified and the total number of protrusions as well as lifetime of each individual protrusion was determined for cells in (A) low, (B) intermediate, and (C) high stiffness matrices (scale bars, 50 μm).

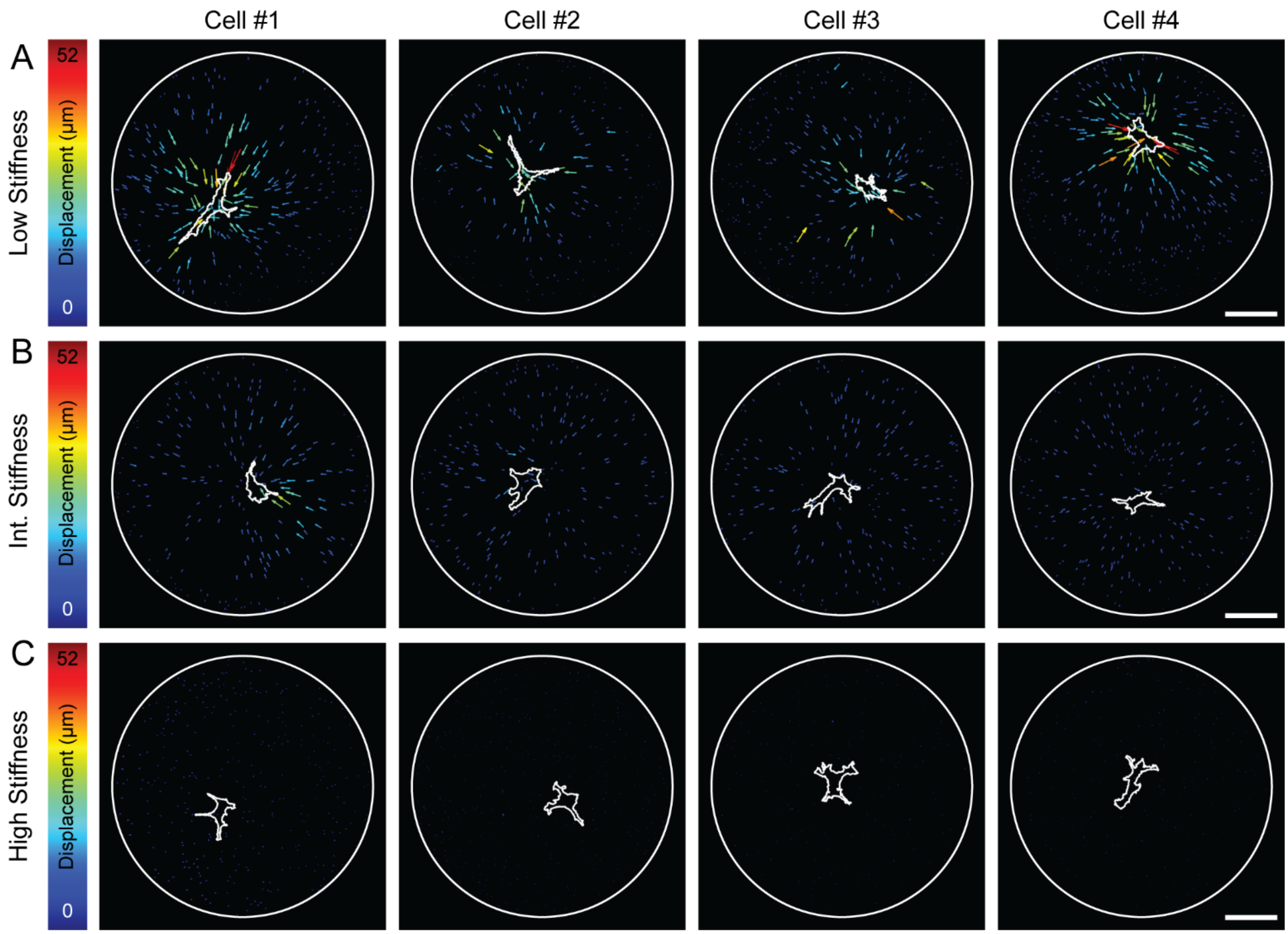

Figure S6. Bead displacement vector plots as a function of matrix stiffness. Representative endothelial cells (cell periphery denoted by white outline) and their respective bead displacements over 12-hours of culture displayed as vectors with magnitude coded by vector size and color in (A) low, (B) intermediate, and (C) high stiffness DexMA matrices (scale bars, 100 μm).

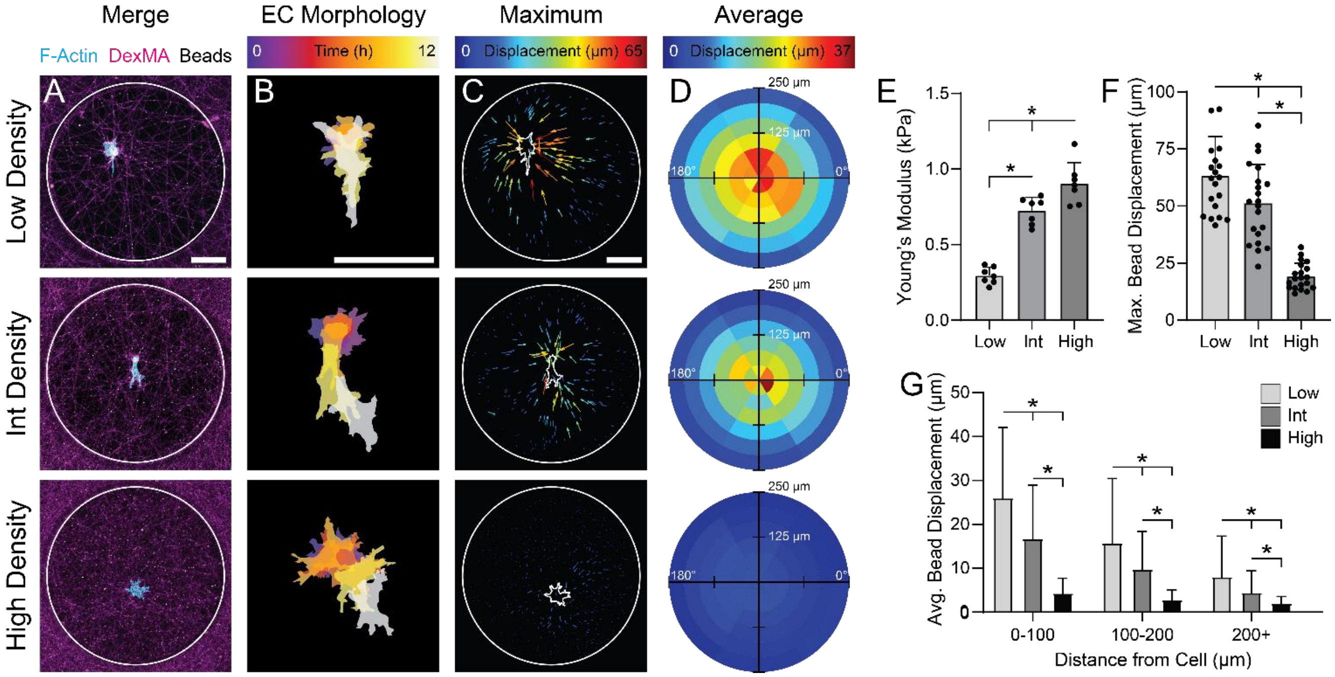

Figure S7. Single endothelial cell morphology and force transmission as a function of fiber density. (A) Representative confocal fluorescent image of phalloidin-stained ECs (cyan), rhodamine-labeled DexMA fibers (magenta), and fiber-embedded fluorescent beads (white) (scale bar, 100 µm). (B) Temporally color-coded overlay of EC cell bodies over a 12 hour time course following initial patterning (scale bar, 100 µm). (C) Maximum displacement of each bead coded by vector length and color over the 12 hour time course (scale bar, 100 µm). (D) Binned average bead displacements color-coded by magnitude for all analyzed ECs with respect to their long axis (0°) (n > 20 cells/group). (E) Young's modulus of DexMA fiber matrices as a function of matrix density (n = 6). (F) Quantification of maximum bead displacement and (G) binned average bead displacements as a function of starting distance from the cell centroid for low, intermediate, and high fiber densities. All data presented as mean ± SD with superimposed data points; asterisk denotes significance with $P < 0.05$.

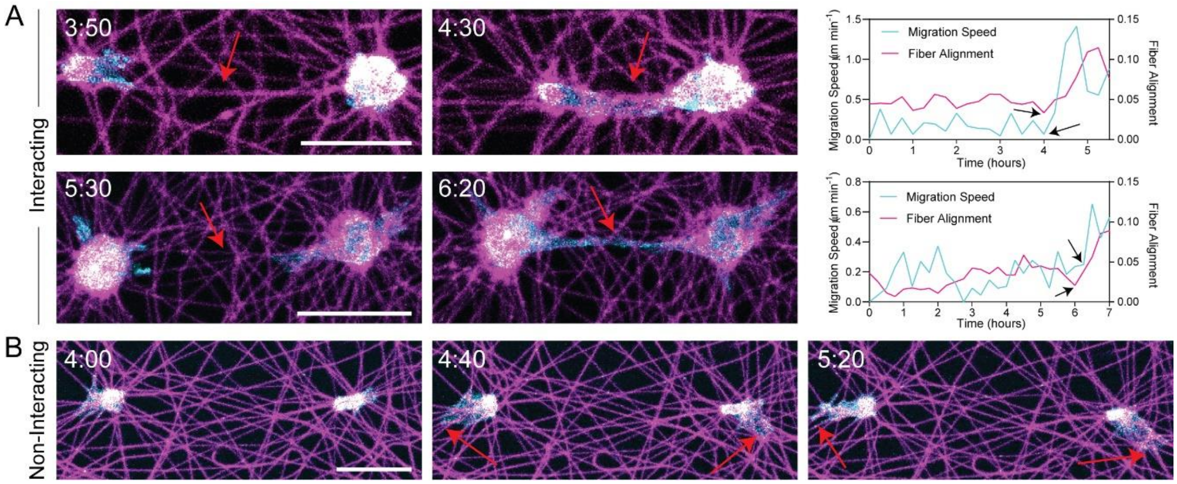

Figure S8. Representative examples of interacting and non-interacting endothelial cell pairs in low stiffness, non-aligned fibrous matrices. (A) Representative confocal fluorescent images of phalloidin-stained ECs (cyan) and rhodamine-labeled fibers (magenta) with quantification of fiber alignment spanning cells and average migration speed of both ECs as a function of time. Red arrows indicate local regions of fiber alignment between neighboring cells (scale bars, 50 µm). (B) Representative confocal fluorescent images of non-interacting ECs. Red arrows indicate cell protrusions extending in the opposite direction of neighboring cell (scale bar, 50 µm).

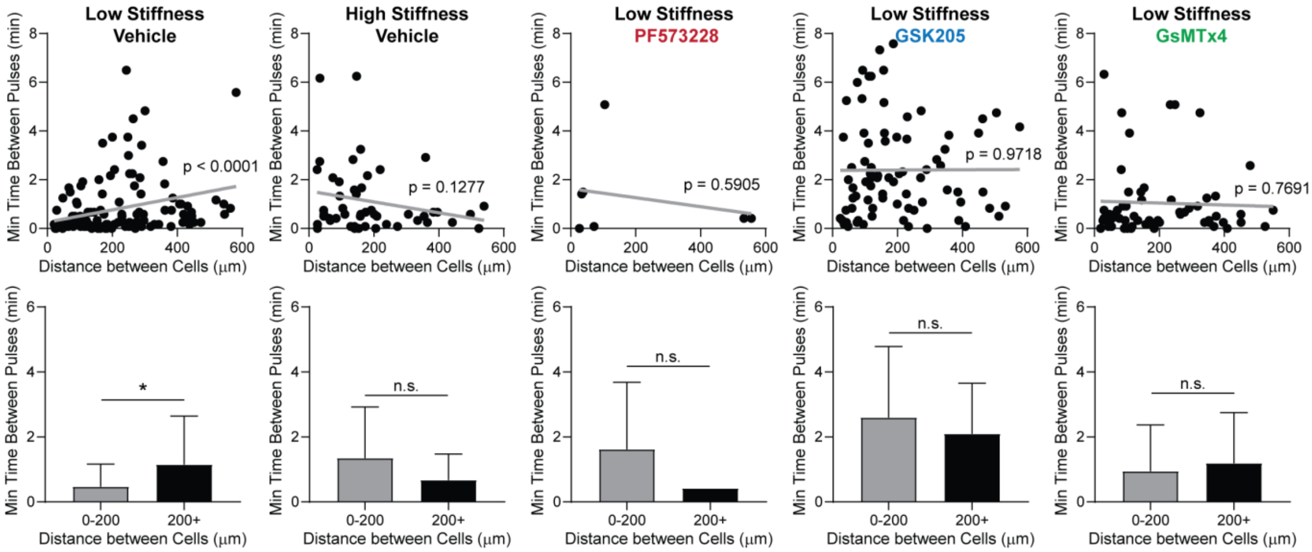

Figure S9. Spatiotemporal Ca^{2+} signaling analysis. Quantification of the minimum time between Ca^{2+} pulses as a function of intercellular distance within EC lines patterned on low stiffness matrices or high stiffness matrices (vehicle control), or low stiffness matrices treated with 10 μM PF573228, 10 μM GSK205, and or 5 μm GsMTx4.

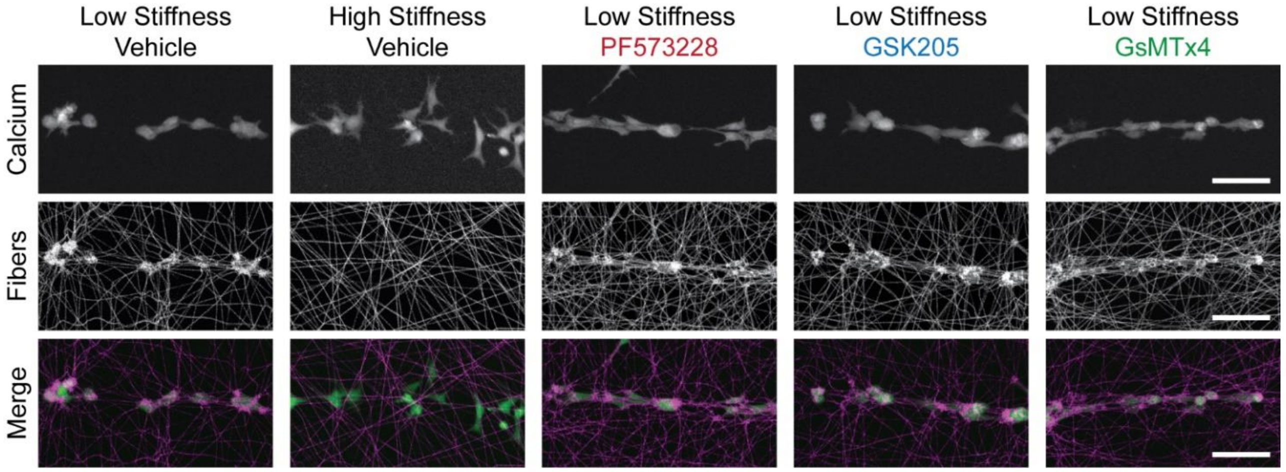

Figure S10. Cell force-mediated matrix deformations following treatment with FAK, TRPV4, and Piezo1 inhibitors. Representative maximum intensity projections of calcium-labeled ECs (green) and rhodamine-labeled fibers (magenta) 2 h after cell line patterning as a function of matrix stiffness and presence of inhibitors confirms that inhibition of FAK (PF573228), TRPV4 (GSK205), or Piezo1 (GsMTx4) does not diminish cell force-mediated matrix reorganization (scale bars: 100 μm).

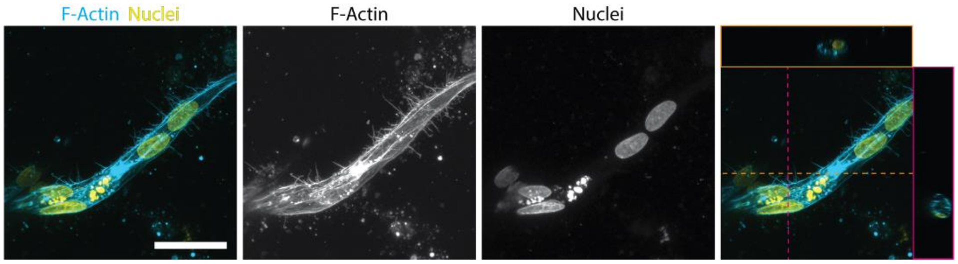

Figure S11. Multicellular clusters of ECs in 3D fibrin possess lumens. Representative confocal maximum intensity projection with orthogonal views (right side) of phalloidin-stained ECs (cyan) and DAPI-stained nuclei (yellow) lumenal structures (location of orthogonal views (orange and magenta images) shown with corresponding dashed lines) in 5.0 mg/ml fibrin hydrogel (scale bar: 50 μm).

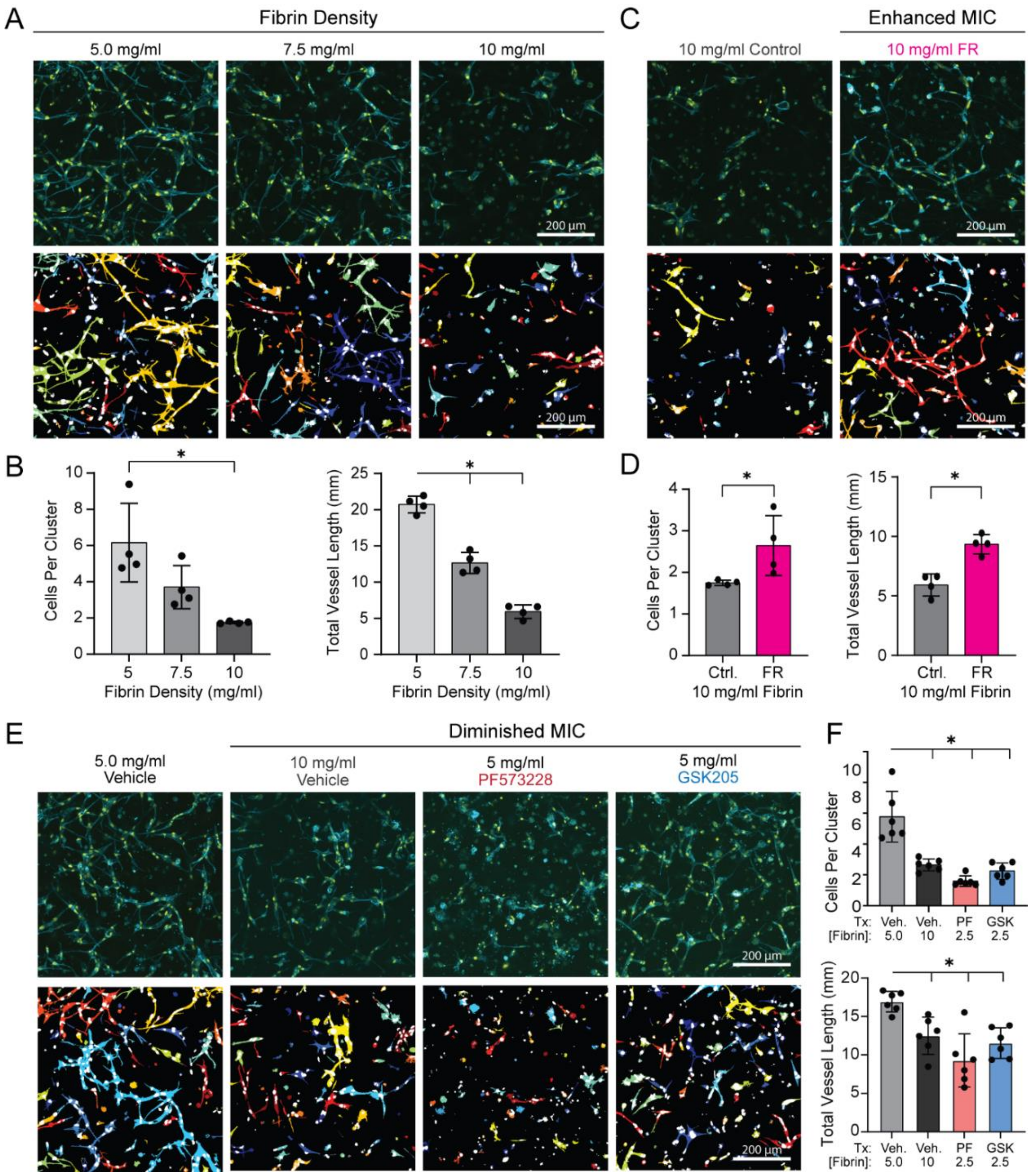

Figure S12. Pharmacologic and biomaterial control of MIC regulates 3D vascular network formation of human lung microvascular endothelial cells in fibrin hydrogels. (A) Representative confocal maximum intensity projections (100 µm thick z-stack) of phalloidin-stained ECs (cyan) and nuclei (yellow) with respective color-coded maps of contiguous actin clusters as a function of fibrinogen concentration. (B) Quantification of total vessel length and average number of cells in each 3D contiguous actin clusters (n = 4 fields of view). (C) Representative confocal maximum intensity projections (100 µm thick z-stack) of phalloidin-stained ECs (cyan) and nuclei (yellow) with respective color-coded maps of contiguous actin clusters in a control and DexVS reinforced fibrin hydrogel. (D) Quantification of total vessel length and average number of cells in each 3D contiguous actin clusters (n = 4 fields of view) shown in C. (E) Representative confocal maximum intensity projections (100 µm thick z-stack) of phalloidin-stained ECs (cyan) and nuclei (yellow) with respective color-coded maps of contiguous actin clusters with cells treated with PF573228 (FAK inhibitor) and GSK205 (TRPV4 inhibitor). (F) Quantification of total vessel length and average number of cells in each 3D contiguous actin clusters (n = 6 fields of view). All data presented as mean ± SD with superimposed data points; asterisk denotes significance with $p < 0.05$.

SUPPLEMENTAL MOVIE CAPTIONS

Movie S1: EC bulk seeding in fibrous DexMA matrices. Representative confocal fluorescence 12-hour timelapse movie of lifeAct-GFP expressing ECs (cyan) and rhodamine-labeled DexMA fibers (magenta) seeded at 250 cells mm^{-2} in low stiffness, cell-deformable and high stiffness, non-deformable matrices (Scale bar: 100 μm).

Movie S2: Single cell patterning in fibrous DexMA matrices with variable matrix stiffness. Representative confocal fluorescence 12-hour timelapse movie of a single lifeAct-GFP expressing EC (cyan), rhodamine-labeled DexMA fibers (magenta), and embedded fluorescent beads (white) patterned in low stiffness, cell-deformable and high stiffness, non-deformable matrices (scale bar: 100 μm).

Movie S3: Single cell patterning in synthetic fibrous DexMA matrices with variable matrix density. Representative confocal fluorescence 12-hour timelapse movie of a single lifeAct-GFP expressing EC (cyan), rhodamine-labeled DexMA fibers (magenta), and embedded fluorescent beads (white) patterned in low and high density matrices with crosslinking equivalent to the lowest stiffness condition (scale bar: 100 μm).

Movie S4: Cell pair patterning in non-aligned fibrous DexMA matrices with variable matrix stiffness. Representative confocal fluorescence 12-hour timelapse movie of pairs of lifeAct-GFP expressing ECs (cyan) and rhodamine-labeled DexMA fibers (magenta) patterned in low stiffness, cell-deformable and high stiffness, non-deformable non-aligned matrices (scale bar: 200 μm).

Movie S5: Cell pair patterning in aligned fibrous DexMA matrices with variable matrix stiffness. Representative confocal fluorescence 12-hour timelapse movie of pairs of lifeAct-GFP expressing ECs (cyan) and rhodamine-labeled DexMA fibers (magenta) patterned in low stiffness, cell-deformable and high stiffness, non-deformable aligned matrices (scale bar: 200 μm).

Movie S6: Calcium transients of ECs in fibrous DexMA matrices with variable matrix stiffness. Representative confocal fluorescence 10-minute timelapse movie of calcium signaling (green) in ECs patterned into multicellular lines in low stiffness, cell-deformable and high stiffness, non-deformable matrices (scale bar: 100 μm).

Movie S7: Calcium transients of ECs in fibrous DexMA matrices in the presence of FAK, TRPV4, and Piezo1 inhibitors. Representative confocal fluorescence 10-minute timelapse movie of calcium signaling (green) in ECs patterned into multicellular lines in low stiffness, cell-deformable matrices with inhibition of FAK (PF573228, 10 μM), TRPV4 (GSK205, 10 μM), and Piezo1 (GsMTx4, 5 μM) (scale bar: 100 μm).

Movie S8: Three-dimensional image stack of EC networks in fibrin hydrogels with variable matrix density, FAK inhibition, and TRPV4 inhibition. Representative confocal fluorescence image stacks (100 μm thick) of phalloidin-stained ECs (cyan) and nuclei (yellow) in 2.5 mg mL^{-1} fibrin, 5.0 mg mL^{-1} fibrin, and 2.5 mg mL^{-1} fibrin with inhibition of FAK (PF573228, 10 μM) or TRPV4 (GSK205, 10 μM) (scale bar: 100 μm).
